# Characterizing proximal risk for depressive symptoms and suicidal ideation with acute cannabis use and withdrawal among adolescents using ecological momentary assessment: Study protocol

**DOI:** 10.1371/journal.pone.0338790

**Published:** 2025-12-18

**Authors:** Isabella Feibus, Marta Borrego Mahiques, Meghan Costello, Kevin Potter, Kate H. Bentley, Bettina B. Hoeppner, Luwei Liu, Bryn Evohr, Lynn Yan, Jodi Gilman, A. Eden Evins, Joe Kossowsky, Randi M. Schuster

**Affiliations:** 1 Department of Psychiatry, Massachusetts General Hospital, Boston, Massachusetts, United States of America; 2 Department of Psychiatry, Harvard Medical School, Boston, Massachusetts, United States of America; 3 Department of Anesthesiology, Critical Care and Pain Medicine, Boston Children’s Hospital, Boston, Massachusetts, United States of America; 4 Department of Anesthesia, Harvard Medical School, Boston, Massachusetts, United States of America; 5 Division of Sleep Medicine, Harvard Medical School, Boston, Massachusetts, United States of America; PLOS ONE, UNITED KINGDOM OF GREAT BRITAIN AND NORTHERN IRELAND

## Abstract

**Background:**

Heavy cannabis use often co-occurs with symptoms of depression in adolescents. Adolescents with past-year cannabis use are at increased risk of major depressive episodes and cannabis use is more than twice as common in adolescents with depression compared to those without. This study seeks to disentangle relationships between acute cannabis use, withdrawal, and abstinence on proximal depression and suicide risk using ecological momentary assessment (EMA) within the context of a randomized controlled trial comparing the effects of cannabis abstinence to cannabis use as usual.

**Methods:**

This study will enroll 200 adolescents aged 12–18 who have daily or near-daily cannabis use and current depressive symptoms. Participants will complete 12 study visits over a 10-week period. At visit 2, participants will be randomized (1:1) to either 8 weeks of sustained cannabis abstinence encouraged through contingency management (CB-Abst) or a monitoring control group (CB-Mon). All participants will complete self-report and interview-based measures of substance use and symptom severity, along with 3 phases of EMA data collection (4 total weeks), involving 8 daily surveys that inquire about past-hour substance use, mood, and situational context. Phases will take place during baseline cannabis use, immediately after randomized abstinence, and following 8 weeks of sustained abstinence. The primary outcomes will be EMA-measured depressive symptoms and suicidal ideation across the 3 EMA phases, by randomized group. Exploratory outcomes will include Fitbit-derived measures of sleep, circadian rhythms, and step count.

**Discussion:**

This is the first study, to our knowledge, to evaluate the acute effects of cannabis use, withdrawal, and sustained abstinence on mood among adolescents with heavy cannabis use and co-morbid symptoms of depression. Results will characterize periods of risk for fluctuations in mood and suicidal ideation across the withdrawal period and aim to guide intervention development to reduce depression and suicide risk among adolescents.

**Trial registration:**

ClinicalTrials.gov NCT06576076

## Introduction

### Background and rationale

Cannabis is one of the most commonly used substances among adolescents in the United States, with about 1 in 3 reporting lifetime cannabis use and 1 in 8 daily or near-daily cannabis use by 12th grade [1]. Adolescence is the period with the highest risk for starting and escalating cannabis use, as well as the emergence of co-morbid mental health issues, including depression and suicidal thoughts. Due to targeted advertising, cannabis has become more widespread and accessible [2], increasing the risk for adolescents to start using potent cannabis products earlier and more often [3]. Previous research has shown a pattern of links between cannabis use and mental health symptoms [4]. This study aims to expand on this research by examining *short-term* changes in mood symptoms in relation to changes in cannabis use during adolescence.

Adolescent depression, suicidal thoughts, and suicide attempts have increased significantly in recent years, with 1 in 6 adolescents experiencing depression, 1 in 5 reporting suicidal thoughts, and 1 in 11 attempting suicide [5,6]. Suicide is the second leading cause of death for adolescents aged 10–24, with rates of suicide attempts and completions rising sharply following the COVID-19 pandemic [7]. As these rates stay high, it is important to consider external factors that may affect mood symptoms, such as cannabis use [8]. Cannabis use is more than twice as common among adolescents with depression compared to those without, and depressed adolescents report more rapid increases in their cannabis use frequency than those without depression [9]. Adolescents who used cannabis in the past year have 2–4 times higher risk for major depressive episodes and suicidal thoughts [4].

Multiple mechanisms have been suggested that could create or sustain this connection between psychological distress and cannabis use. For some adolescents, cannabis use might be maintained by negative reinforcement to the extent that cannabis provides an immediate but not lasting reduction in depressive symptoms [10,11]. Additionally, cannabis could be a factor in the complex causes of depression and suicidal thoughts, with 1 in 14 cases of adolescent depression likely linked to cannabis use [8]. Adolescent cannabis use consistently predicts higher chances of developing depression and suicidal thoughts in young adulthood [4], and these links are strongest among those with earlier onset and more frequent use during adolescence [12]. Substance use and mental health issues identified in adolescence often lead to ongoing problems with substance use and mental health into adulthood [13], making it important to support youth well-being for both immediate and future benefits.

Although there is strong evidence that cannabis use is a distal risk factor for depression, proximal effects of cannabis use, withdrawal, and abstinence on mood have not been studied. To make an impact on rates of depression and suicide, it is essential to understand how cannabis acutely influences mood and suicidal thoughts in depressed adolescents who heavily use cannabis. This study aims to determine whether, when, and for whom cannabis use affects the risk of adverse mood and suicide outcomes, as well as to identify factors that may sustain use (such as temporary relief from distress) in this group.

### Objectives and Hypotheses

We will conduct a 10-week, multi-method, randomized trial using a 2-arm within-subject parallel group design. Participants will be randomized after a 2-week baseline period of cannabis use as usual to either 1) 8 weeks of sustained cannabis abstinence via contingency management (CB-Abst) or 2) a monitoring control group maintaining usual cannabis use (CB-Mon). The study will include 3 ecological momentary assessment (EMA) phases to assess how mood varies during cannabis use, withdrawal, and after 8 weeks of sustained abstinence.

*Aim 1*: We will evaluate a model of cannabis use in which negative mood and suicidal ideation precipitate cannabis use events and are temporarily alleviated and stabilized through acute cannabis use (i.e., negative reinforcement). We will analyze EMA responses in the entire sample during a phase of usual cannabis use before randomization (EMA Phase 1).

*Hypothesis 1.1:* Negative mood will predict same-day cannabis use events and higher self-reported motivation to use cannabis for mood improvement.

*Hypothesis 1.2:* Suicidal ideation reported within 1 hour after cannabis use will be less intense and less variable than mood and suicidal ideation reported during same-day random-prompt EMA reports occurring at least 1 hour since last cannabis use.

*Aim 2:* We will examine whether negative mood and suicidal ideation decrease in overall (mean) levels and variability after 8 weeks of sustained cannabis abstinence. To this end, we will compare EMA responses during EMA Phase 2 (Randomized Withdrawal) and 3 (Randomized Sustained Abstinence) among CB-Abst versus CB-Mon.

*Hypothesis 2.1:* Among adolescents randomized to cannabis abstinence (CB-Abst), but not those randomized to maintained cannabis use as usual (CB-Mon), negative mood and suicidal ideation reported during random prompt EMA reports will increase and become more variable during the first week of cannabis withdrawal (EMA Phase 2).

*Hypothesis 2.2:* Among participants randomized to cannabis abstinence (CB-Abst), negative mood and suicidal ideation will be lower after 8 weeks of sustained abstinence (EMA Phase 3) compared to negative mood and suicidal ideation during baseline cannabis use as usual (EMA Phase 1) and during cannabis withdrawal (EMA Phase 2). There will be no difference in the intensity or variability of mood or suicidal ideation across the study for participants randomized to a monitoring condition with maintained cannabis use as usual (CB-Mon).

## Methods

### Participants, interventions, and outcomes

#### Design.

**Fig 1:**
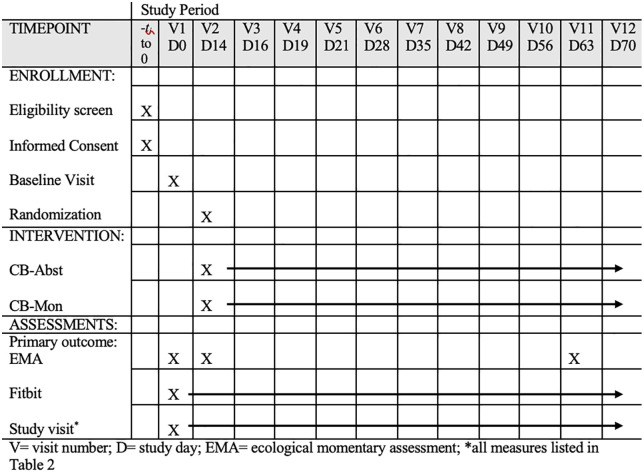
SPIRIT schedule of enrollment, interventions, and assessment.

**Fig 2:**
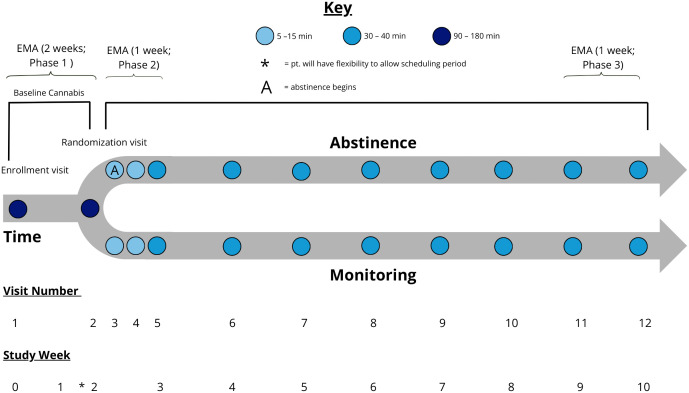
Visual representation of study schema.

Participants will be enrolled in a 3-phase, single-blinded protocol (see Fig 1) and randomly assigned to either 8 weeks of abstinence with contingency management (CB-Abst; n = 100) or non-contingent monitoring with no abstinence requirement (CB-Mon; n = 100). They will complete 12 study visits and 3 phases of EMA surveys over 10 weeks. Study visits will occur at baseline (V1), approximately 2 weeks later (V2), and weekly throughout the trial (V5-12). Additionally, there will be 2 brief visits in the first week post-randomization mainly for abstinence verification (V3 and V4). Visits will take place in a private room at the participant’s school or local library, in lab settings, or virtually via video conferencing. Participants will also provide sleep and activity data via a Fitbit Inspire 3, which they will wear continuously from enrollment at Visit 1 through Visit 12 (around 70 days). This study has been registered on ClinicalTrials.gov (NCT06576076). Refer to Figs 1 and 2 for a SPIRIT schedule of enrollment and a study schema overview and S1 file for a completed SPIRIT checklist.

### Participants

Participants will be included if they are aged 12−18 years (inclusive); have current daily or near-daily cannabis use (i.e., use ≥ 4 days per week on average); score ≥ 5 on the Patient Health Questionnaire-9 (PHQ-9) [14] or currently have a depressive episode (Mini International Neuropsychiatric Interview Kid 7.0.2 [15]). Additional inclusion criteria include access to an internet-capable smartphone; providing of at least 1 collateral contact for emergency situations; providing informed assent (or consent if 18 years) and parent/guardian consent if under 18 years; greater than approximately 50% response rate to EMA prompts during the first EMA phase (Baseline Use as Usual); having no immediate plan to stop cannabis use in the next three months; and testing positive for cannabis on baseline urinalysis.

Exclusion criteria will include any factor that impairs the ability to understand and participate effectively, such as acute intoxication at the time of consent; cannabis use exceeding 4 times per day on average; inability to speak or write English fluently; severe cognitive impairment; a current diagnosis of epilepsy; individuals under legal protection of the government or state (wards of the state); and a lack of acknowledgment that EMA survey responses are not reviewed in real time. Participants must acknowledge that responses are monitored (but not in real time) by clinical staff for safety, and phone-based outreach, risk assessment, or safety interventions by study staff clinicians may occur within 24 hours if high intent to act on suicidal thoughts is reported.

### Randomization

Once enrolled and after the baseline phase (EMA Phase 1; Cannabis Use as Usual), participants will complete a randomization visit (Visit 2). Participants will be randomly assigned to either 8 weeks of abstinence with contingency management (CB-Abst: n = 100) or non-contingent monitoring with no abstinence requirement (CB-Mon: n = 100), using a 1:1 ratio in blocks of 6. Randomization will be stratified by age (<=14 years old vs >=15 years old), sex (assigned male vs female at birth), and depression severity (mild/moderate vs. moderately severe/severe [14]).

### Interventions

#### Abstinence.

Participants assigned to the abstinence condition (CB-Abst) will be incentivized through contingency management, as described in previous studies [16–18]. They will be reimbursed via a two-track incentive system that includes static payments for attendance of study visits and increasing payments for abstinence, along with bonus payments for meeting EMA compliance thresholds of 50% or 80% in each of the 3 EMA phases (Table 1). During the baseline visit (Visit 1), participants will review an abstinence contract (S2 File) with a study staff member, which details expected behavior changes and the payment schedule [17]. This contract will be reviewed with all participants at the baseline visit to set expectations and confirm their willingness to abstain from cannabis use. At the second visit, participants randomized to CB-Abst will be asked to stop cannabis use immediately for the remainder of the study and will complete an abstinence planning sheet (S3 File) with study staff.

**Table 1:** Schedule of remuneration.

Study Visit Payments				
	CB-Mon		CB-Abst	
Visit Number	Attendance	Abstinence	Attendance	Abstinence
1 Enrollment	$10	N/A	$10	N/A
2 Randomization	$10	N/A	$10	N/A
3 Day 16	$20	N/A	$5	$15
4 Day 19	$25	N/A	$5	$20
5 Week 3	$30	N/A	$5	$25
6 Week 4	$35	N/A	$5	$30
7 Week 5	$40	N/A	$5	$35
8 Week 6	$45	N/A	$5	$40
9 Week 7	$50	N/A	$5	$45
10 Week 8	$55	N/A	$5	$50
11 Week 9	$60	N/A	$5	$55
12 Week 10	$65	N/A	$5	$65
Max Subtotal	$445		$70	$375
**Compliance Payment (CB-Mon and CB-Abst)**				
Phase 1 (Baseline Use as Usual)				
EMA Compliance (approximately 50% completion or 80% completion)				$25 or $100, respectively
Fitbit Compliance Phase 1 (66% completion)				$25
Phase 2 (Randomized Withdrawal)				
EMA Compliance (approximately 50% completion or 80% completion)				$25 or $100, respectively
Fitbit Compliance Phase 2 (66% completion)				$25
Phase 3 (Randomized Sustained Abstinence)				
EMA Compliance (approximately 50% completion or 80% completion)				$25 or $100, respectively
Fitbit Compliance Phase 3 (66% completion)				$25
Overall Fitbit and EMA Bonus (completion across whole study)				$50
**Max total earnings $870**				

Abstinence in the CB-Abst group will be confirmed through quantitative urinary levels of 11-nor-9-carboxy-Δ9-tetrahydrocannabinol (THCCOOH), the main secondary metabolite of THC and a widely accepted biomarker for cannabis use testing. Participants who resume cannabis use within the first week of abstinence will be allowed one opportunity to restart the abstinence protocol and stay in the study. Participants who resume cannabis use again after the first week will continue with data collection but will be excluded from analyses involving CB-Abst participants. See section below for additional details on the protocol for verifying abstinence.

#### Monitoring.

Participants randomized to monitoring (CB-Mon) will not be required by the study to change their cannabis use habits after Visit 2. CB-Mon will receive payments that increase gradually based on session attendance (Table 1). Individuals assigned to monitoring who happen to stop using cannabis or show interest in stopping will not be removed from the study. If they want, these individuals will be given substance use resources at the time they express interest in stopping and again at the end of the study.

### Recruitment and retention

Participants will be recruited from local high schools through annual high school screenings, flyers, and direct outreach to school staff. Additional national recruitment efforts will involve community engagement via online postings and flyers. Recruitment will also be supported by advertisements on social media through BuildClinical, a targeted marketing platform designed to promote study recruitment among specific age groups, as well as through advertisements on Massachusetts public transit.

Study eligibility will be assessed through a telephone screening and confirmed at the baseline visit (Visit 1). Retention will be supported by compensation of up to $870 for attending study visits, achieving biochemically verified abstinence, and complying with Fitbit and EMA protocols (Table 1). Total maximum earnings will be matched across groups to avoid confounding the effects of monetary payments with depressive symptoms and suicidal ideation.

### Measures

Data will be collected through interviews, self-report questionnaires, urine samples for qualitative and quantitative drug testing, and EMA- and Fitbit-based data collection. All measures are listed in Table 2.

**Table 2:** Time and events.

Passive parental consent 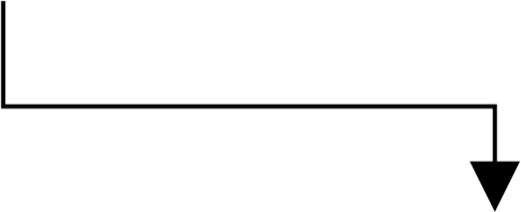	Parent/ participant consent/assent 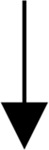			Participants in CM discontinue cannabis use 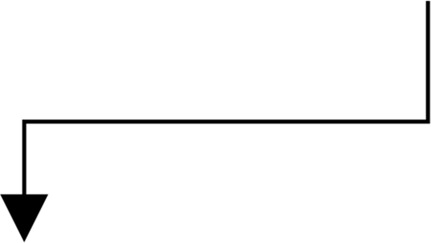										
	**V0**		**BL (V1)**	**V2**	**V3**	**V4**	**V5**	**V6**	**V7**	**V8**	**V9**	**V10**	**V11**	**V12**
Approximate Days Since Baseline			0	14	16	19	21	28	35	42	49	56	63	70
Fitbit Inspire 3			x	x	x	x	x	x	x	x	x	x	x	x
Phone Screener	x													
EMA Assessments (x indicates when EMA assessment phases begin)			x	x									x	
*Substance Use Questionnaires*														
AUDIT			x											
CUDIT-R			x											
CWS			x	x	x	x	x	x	x	x	x	x	x	x
ECDI			x											
MCQ-SF			x	x	x	x	x	x	x	x	x	x	x	x
MMM			x											
TLFB			x	x	x	x	x	x	x	x	x	x	x	x
Substance Use History			x											
*Depressive Symptoms and Suicidal Thoughts and Behaviors*														
BAI			x											x
PHQ-9			x	x	x	x	x	x	x	x	x	x	x	x
C-SSRS			x	x	x	x	x	x	x	x	x	x	x	x
RFL-A			x	x			x	x	x	x	x	x	x	x
SITBI-R			x											
*Other Relevant Confounds and Covariates*														
ASRI			x											x
Adverse Events				x	x	x	x	x	x	x	x	x	x	x
Concomitant Medications			x	x	x	x	x	x	x	x	x	x	x	x
Demographics			x											
Family Psychiatric History				x										
PSQI			x	x			x	x	x	x	x	x	x	x
MCQ Delay Discounting				x										
Medical History			x											
CBCL			x											
MINI (CUD and MDE)			x											
SUPPS-P				x										
*Urinalysis Test*														
Toxicology			x	x	x	x	x	x	x	x	x	x	x	x

AUDIT: Alcohol Use Disorder Identification Test; CUDIT-R: The Cannabis Use Disorder Identification Test – Revised; CWS: Cannabis Withdrawal Scale; ECDI: Penn State Electronic Cigarette Dependence; MCQ-SF: Marijuana Craving Questionnaire- Short Form; MMM: Marijuana Motives Measure; TLFB: Timeline Followback Interview; BAI: Beck Anxiety Inventory; PHQ-9: Patient Health Questionnaire-9; C-SSRS: Columbia Suicide Severity Rating Scale; RFL-A: The Reasons of Living for Adolescents; SITBI-R: Self-injurious Thoughts and Behaviors Interview-Revised; ASRI: Conners ADHD Self-Report Index (ASRI); KSADS: Family Psychiatric History; PSQI: Pittsburgh Sleep Quality Index; MCQ Delay Discounting; CBCL: Child Behavior Checklist Youth Self Report; MINI (CUD and MDE): Mini International Neuropsychiatric Interview (MINI) Kid 7.0.2: Cannabis Use Disorder and Major Depressive Episode; SUPPS-P: Short UPPS-P Impulsive Behavior Scale

### Fitbit

Fitbit Inspire 3 watches will be worn throughout the study to evaluate changes in sleep and activity. Fitbit metrics include sleep duration, sleep fragmentation, sleep timing, sleep timing variability, and physical activity.

### Ecological momentary assessment

Participants will complete 3 phases of EMA data collection: during baseline cannabis use (EMA Phase 1; Cannabis Use as Usual), in the week following randomization (EMA Phase 2; Randomized Withdrawal), and after 8 weeks of sustained cannabis abstinence (EMA Phase 3; Randomized Sustained Abstinence). EMA data will be collected via Metricwire**,** a secure smartphone-based app with full-cycle project capabilities, including real-time observation and reporting.

To optimize response rates, participants can choose between two survey schedules, each with 8 prompts per day. The first schedule features different survey blocks for weekdays and weekends. The second schedule applies the weekend block every day, reducing the chance of receiving survey notifications early in the morning. Weekday survey blocks are: 7:00–8:00am (1 prompt), 8:01am-2:00 pm (1 prompt), 2:01–4:00 pm (2 prompts), 4:01–7:00 pm (2 prompts), 7:01–10:00 pm (2 prompts). Weekend survey blocks are: 7:00–10:00am (1 prompt), 10:01am-2:00 pm (1 prompt), 2:01–4:00 pm (2 prompts), 4:01–7:00 pm (2 prompts), 7:01–10:00 pm (2 prompts). Survey prompts will be randomly sent within each block. Participants will be notified via push notifications. If a participant does not respond within 15 minutes, they will be re-notified. Surveys will stay open for 1 hour.

EMA surveys will utilize short, multiple-choice questions with logic-branched follow-ups to evaluate momentary situational factors. Questions (S4 File) will assess craving to use cannabis (1 [No, not at all] to 100 [Yes, very much]), whether participants are currently using cannabis or have used it within the past hour (including times and amounts since the last EMA survey), and if so, if they used cannabis to improve their mood (1 [Not at all] to 100 [Very much]).

Participants will also be asked about current positive and negative affect and suicidal ideation (1 [Not at all] to 100 [Very much]). Affect items will measure depleted mood (Sad, Self-hatred, Numb, Hopeless, and Fatigued), cognitive impacts (Disinterested and Inattentive), and negative activation (Agitated, Irritated, and Anxious). Suicidal ideation items will evaluate the presence, urge, intensity, and controllability of passive suicidal thoughts (such as thoughts about death, wishing suffering would end, and feeling better off dead), as well as suicidal and non-suicidal self-injury urges. Questions will also evaluate participants’ intent to kill themselves on that given day. If any intent is indicated, additional questions will gather information on participants’ location, activities, social circumstances and setting (e.g., alone or with others; with others using drugs), exposure to cannabis and cannabis use cues, and concurrent use of other substances.

At the baseline visit, all participants will complete a safety plan [19] (S5 file) designed to help manage moments of intense distress and suicidal urges. The safety plan will be uploaded into Metricwire and will be automatically displayed if safety concerns (i.e., participant rates intent to act on suicidal ideation 50/100 or higher) are reported during any EMA prompts.

### Substance use questionnaires

At the baseline visit (V1), participants will complete the **Mini International Neuropsychiatric Interview** (MINI) **Kid 7.0.2** [15]: Cannabis Use Disorder (CUD) module. The MINI Kid is a brief, semi-structured diagnostic interview that evaluates DSM-IV and ICD-10 psychiatric disorders in children and adolescents. The CUD module asks about cannabis-related symptoms over the past year.

The **Alcohol Use Disorder Identification Test** (AUDIT) [20], [21] is a 10-item screening tool that assesses alcohol consumption, drinking behaviors, and alcohol-related problems over the past year. Participants will complete a self-report survey during the baseline visit (V1).

The **Cannabis Use Disorder Identification Test-Revised** (CUDIT-R) [22] is an 8-item screening tool that assesses cannabis use, related behaviors and consequences, and symptoms of cannabis dependence over the past 6 months. Participants will complete a self-report survey during the baseline visit (V1).

The **Cannabis Withdrawal Scale** (CWS) [23] is a 26-item screening tool that measures the current intensity of cannabis withdrawal symptoms using a 10-point Likert scale, ranging from 1 (“not at all”) to 10 (“extremely”). Participants will complete this self-report survey at all visits (V1-V12).

The **Penn State Electronic Cigarette Dependence Index** (ECDI) [24] is a 10-item screening tool evaluating electronic cigarette dependence. Participants will complete a self-report survey at the baseline visit (V1).

The **Marijuana Craving Questionnaire- Short Form** (MCQ-SF) [25] is a 12-statement screening tool assessing current thoughts and feelings about smoking marijuana across four subscales: compulsivity, emotionality, expectancy, and purposefulness. Participants will complete this self-report survey at all visits (V1-V12).

The **Marijuana Motives Measure** (MMM) [26] is a 25-item screening tool assessing motives for use of marijuana by measuring five domains of motivating factors for marijuana use: enhancement, conformity, expansion, coping, and social motives. Participants will complete this survey at the baseline visit (V1).

Participants will complete a **Timeline Followback** (TLFB) [27] interview assessing the frequency, quantity, and duration of cannabis, nicotine, alcohol, and any other non-medical substance use. A 90-day recall period will be queried at the baseline visit (V1), and the time elapsed since the previous visit will be queried at V2-12.

At the baseline visit, participants will complete a **Substance Use History** interview assessing lifetime history with cannabis, nicotine, alcohol, and any other non-medical substance use. For any substance use endorsed, participants will provide age of first use and frequency of past-year and 90-day use.

### Depressive symptoms and suicidal thoughts and behaviors

At the baseline visit (V1), participants will complete the **Mini International Neuropsychiatric Interview** (MINI) **Kid 7.0.2** [15]: Major Depressive Episode (MDE) module. The MDE module includes two screening questions for both lifetime and current depression symptoms; if these questions are answered affirmatively, additional follow-up questions specific to the relevant time period are asked.

The **Child Behavior Checklist- Youth Self-Report** (CBCL-YSR) [28] is self-report survey assessing internalizing and externalizing problematic behaviors. The measure assesses “Total Competency,” which is a scale comprised of competency in activities, social functioning, and school performance. Participants will complete this survey at the baseline visit (V1).

The **Beck Anxiety Inventory** (BAI) [29] is a 21-item inventory measuring the severity of common symptoms of anxiety in the past month. Participants will complete this self-report survey at the baseline and final study visit (V1 and V12).

The **Patient Health Questionnaire-9** (PHQ-9) [14] is a self-administered version of the PRIME-MD diagnostic instrument for common mental disorders. Participants rate how often they have been bothered by the 9 criteria on which the diagnosis of DSM-IV depressive disorders is based. Participants will complete this self-report survey at all visits, with a past 2-week period assessed at the baseline visit (V1) and a past week period assessed at all follow-up visits (V2-12).

The **Columbia-Suicide Severity Rating Scale** (C-SSRS) [30] interview evaluates the severity of suicidal ideation on a 5-point ordinal scale (1 = wish to be dead, 2 = nonspecific active suicidal thoughts, 3 = suicidal thoughts and methods, 4 = suicidal intent, and 5 = suicidal intent with a plan) and rates suicidal behaviors on a nominal scale that includes actual, aborted, and interrupted attempts; preparatory behaviors; and non-suicidal self-injurious actions. At the baseline visit (V1), participants will be asked about past-month suicidal ideation and past 3-month suicidal behaviors. During all follow-up visits (V2-12), ideation and behaviors will be assessed since the previous visit.

The **Reasons for Living for Adolescents** (RFL-A) [31] is a 32-item screening tool assessing risk for committing suicide by measuring reasons for living across five subscales: family alliance, suicide-related concerns, self-acceptance, peer acceptance and support, and future optimism. Participants will complete this survey at V1, V2, V5-V12.

The **Self-injurious Thoughts and Behaviors Interview-Revised (SITBI-R)** [32] is a semi structured interview assessing the presence, frequency, and characteristics of five types of self-injurious thoughts and behaviors: suicidal ideation, suicidal plans, suicidal gestures, suicidal attempts (including aborted and interrupted), and non-suicidal self-injury. Participants will complete an adapted version of this interview with select questions removed to reduce participant burden at the baseline visit (V1).

### Relevant confounds and covariates

The **Conners ADHD Self-Report Index** (ASRI) [33] is a 10-item screening tool assessing past-month severity of ADHD symptoms. Participants will complete this self-report survey at V1 and V12.

**Adverse Events** will be assessed at V2-V12. Participants will be asked if they have experienced any changes in their mental or physical health since their last visit. If participants have a significant increase in scores on the C-SSRS (≥ 1) or on the PHQ-9 (≥5), this will be recorded as an adverse event.

**Concomitant Medications** will be assessed at every visit. At the baseline visit (V1), participants will report any medications they regularly take including the name, indication, dose, frequency, route of administration, and date started. At all follow-up visits (V2-12), participants will report if they have had medication changes since the last visit.

At the baseline visit (V1), participants will complete a **demographics** survey including characteristics such as sex, gender identity, sexuality, race, education status, average grades in school, estimated household income, height, and weight.

At the second visit (V2), participants will complete a **Family Psychiatric History** interview. This interview is adapted from the Family History for Biological Relatives module of the K-SADS [34].

The **Pittsburgh Sleep Quality Index** (PSQI) [35] is 19-item screening tool assessing seven facets of sleep quality and disturbance, including subjective sleep quality, sleep latency, sleep duration, habitual sleep efficiency, sleep disturbance, use of sleeping medication, and daytime dysfunction. Participants will complete this self-report survey at V1, V2, V5-V12.

The **Monetary-Choice Questionnaire Delay Discounting** (MCQ) [36] is a 27-item screening tool that asks participants to choose between a smaller, immediate monetary reward and a larger, delayed monetary reward. Participants will complete this self-report survey at their second visit (V2).

At the baseline visits, participants will complete a **medical history check** asking if they have any significant or uncontrolled medical conditions.

The **Short UPPS-P Impulsive Behavior Scale** (SUPPS-P) [37] is a 20-item screening tool assessing impulsive behavior by measuring 5 subscales: negative urgency, premeditation, perseverance, sensation seeking, and positive urgency. Participants will complete this self-report survey at their second visit (V2).

### Verification of abstinence

Urine samples will be collected on the day of each study visit for all participants. Qualitative urine drug testing will be performed using the Medimpex Multi Drug 10 Panel Drug Test, which will detect recent exposure to cocaine, THC, opiates, amphetamines, methamphetamine, phencyclidine, benzodiazepines, barbiturates, methadone, and oxycodone. Dominion Diagnostics (Kingstown, RI, USA) will analyze the urine samples with liquid chromatography/tandem mass spectrometry (LC-MS/MS) to measure the concentration of creatinine-adjusted 11-nor-9-carboxy-Δ^9^-tetrahydrocannabinol (THCCOOH). Self-reported cannabis abstinence will be biochemically confirmed in the CB-Abst group by progressively decreasing urine concentrations of THCCOOH using a statistical model [38].

### Primary outcomes

Primary outcomes include EMA-derived measures of depressive symptoms and suicidal ideation recorded during baseline cannabis use (EMA Phase 1), 1 week (EMA Phase 2), and 8 weeks after randomization (EMA Phase 3). All items are rated from 0 – No, not at all to 100 – Yes, very much.

### Consent or assent

Participants considered potentially eligible through the telephone screening will give written consent (assent if under 18) after study staff explain the study, including possible risks, the right to withdraw, and confidentiality details. Informed consent procedures will be performed with a parent or guardian of participants under 18 before starting any study procedures. A licensed clinician will obtain informed consent or assent before administering any study procedures.

### Blinding

The group assignment will be blinded to outcome assessors, investigators, and analysts. Staff who are unblinded will handle contingency management procedures, perform urinalysis, calculate abstinence based on urinalysis results, process payments, and monitor survey compliance. An analyst-blind approach will be used during the creation of analysis scripts to keep analyses confirmatory [39]. Data initially provided to the analyst will have the assignments randomly shuffled to ensure that any unanticipated changes to the original analytic plan are not unconsciously influenced by outcomes that produce significant findings.

## Methods

### Data collection, management and analysis

#### Data collection methods and management.

EMA data will be collected through Metricwire, and all data will be stored anonymously with a unique random ID,. REDCap questionnaires administered during study visits will be automatically and securely stored on a SQL Server, accessed via industry-standard TLS 256-bit RSA encryption during data transfers. After each visit, study staff will perform quality checks on the data to ensure completeness and accuracy.

Fitbit data will be processed in a customized data management environment that interfaces with Fitbit cloud servers. This platform offers secure tools for data collection and management, enabling remote data gathering without needing to return devices for data extraction. Sleep-wake metrics will be generated from signals collected by accelerometry, heart rate, and heart rate variability, processed by Fitbit’s proprietary, validated algorithm [40]. Valid sleep data will be defined as non-nap sleep durations exceeding 3 hours. Physical activity will be estimated from daily step counts detected by Fitbit’s triaxial accelerometer, which aligns with direct observation [41], and will be reported as daily and intraday (per minute) step counts. From step counts, we will calculate the minutes spent in sedentary, light, moderate, and vigorous activity. Metrics will be computed for each 24-hour period, generating averages and within-subject standard deviations to evaluate variability.

### Sample size

We plan to recruit 200 participants. The R package ‘EMAtools’ (version 0.1.3) [42] was used to calculate the power for the planned analyses. Preliminary analyses from a pilot study of this protocol found that effect size estimates for significant effects ranged from 0.19 to 0.44, and 77% of participants completed all study phases, with an average of 76% of prompts completed. Power estimates were therefore calculated to detect a small effect (a Cohen’s d of 0.2) using linear multi-level models with a nesting structure for the proposed EMA analyses and an intra-class correlation of.09.

### Statistical methods and analyses

#### Outcomes.

We will test hypotheses across 7 outcomes. Negative mood is measured using 3 composite scores, and suicidal ideation with 1 composite score plus 2 individual EMA items: 1) the average of 5 items assessing depleted mood (scores for feeling Sad, Self-hatred, Numb, Hopeless, and Fatigued), 2) the average of 2 items measuring negative cognitive impacts (scores for feeling Disinterested and Inattentive), 3) the average of 3 items regarding negative activation (scores for feeling Agitated, Irritated, and Anxious), 4) the average of 3 items assessing passive suicidal thoughts (scores for Thoughts about death, Wishing suffering were over, and Feeling Better off as dead), 5) the individual item for suicidal urges, and 6) the individual item for non-suicidal self-injury urges. All items are rated on a scale from 0 (No, not at all) to 100 (Yes, very much). Additionally, we assess acute cannabis use as a binary variable (1 = used in the hour before the current prompt, 0 = no use in the last hour), which is incorporated into the final outcome: 7) whether cannabis was used in the last hour to improve negative mood, measured on a scale from 0 (No cannabis use) to 100 (Yes, very much).

### Statistical models

Outcomes will be modeled using zero-inflated multilevel beta regression models. We will include predictors in 3 different parts of the statistical model: (1) the mean for the non-zero continuous part, (2) the variance for the non-zero continuous part, and (3) the probability of zero occurrence (the zero component). Additionally, for components 1 (mean) and 3 (zero), we will add participant-varying intercepts to account for individual differences and the repeated measures nature of the EMA data.

All models will include covariates for weekday (categorical, Monday-Sunday), prompt block (categorical, 1–5), age (continuous), and gender (categorical). These covariates will be included in component 1 (mean) for the models.

### Implementation of analyst-blind approach

We will implement our analyst-blind approach for each analysis by randomly shuffling the key contrast (e.g., group randomization to abstinence or monitoring) beforehand. This method will allow flexibility to respond to potential unexpected challenges in data cleaning and modeling (such as autocorrelation in EMA data, convergence issues, and risks of complete separability in the zero-inflation component) while reducing the risk of unconscious bias that could lead to inflating statistical significance. Once data cleaning and modeling decisions are finalized and any changes from the initial proposals are documented, the original data will be analyzed, and confirmatory results will be reported.

### Hypothesis 1.1

Included in analyses will be all participants in EMA Phase 1, the 2-week period before randomization. The main outcome will be the rating of intention to use cannabis to improve mood. We will create separate models for each of the 6 measures of negative affect or suicidal ideation. For each model, we will use 2 continuous predictors: affect/ideation collected during the same prompt (concurrent) and affect/ideation collected during the same day’s previous prompt (lag-1). These predictors will be used in components 1 (mean) and 3 (zero). Hypotheses 1.1 will be supported if either (a) there is a significant link between affect/ideation and increased likelihood of immediate cannabis use (component 3), or (b) there is a significant link between affect/ideation and the intention to use cannabis to improve mood when using (component 1).

### Hypothesis 1.2

Data will be limited to all participants in EMA phase 1 during the 2-week period before randomization. The outcomes of interest will include the 6 affect/ideation measures, with separate models created for each outcome. The main predictor will be a categorical variable for acute cannabis use (cannabis use in the last hour = 1). This predictor will be included in components 1 (mean) and 2 (variance). Hypothesis 1.2 will be supported if acute cannabis use is linked to lower affect/ideation ratings (component 1) and decreased variance (component 2).

### Hypothesis 2.1

Data will include all phases (1–3) from (a) participants randomized to CB-Mon and (b) the data for participants randomized to CB-Abst associated with verified abstinence. The outcomes of interest will be the six affect/ideation measures, with separate models fitted for each outcome. Models will include predictors for group membership (CB-Abst vs. CB-Mon) and phase (Phase 1 vs. 2, Phase 1 vs. 3). Predictors will be included in components 1 (mean) and 2 (variance). The key contrast will compare CB-Abst and CB-Mon during phase 2 (the first week of abstinence). Hypothesis 2.1 will be confirmed if abstinence is associated with higher (worse) affect/ideation ratings (component 1) and increased variance (component 2).

### Hypothesis 2.2

Data will include all phases (1–3), participants randomized to CB-Mon, and the data for participants randomized to CB-Abst with verified abstinence. The outcomes of interest will be the 6 affect/ideation measures, with separate models fitted for each outcome. Models will include predictors for group membership (CB-Abst vs. CB-Mon) and phase (Phase 1 vs. 2, Phase 1 vs. 3). Predictors will be incorporated into components 1 (mean) and 2 (variance). The key contrast will be the differences between CB-Abst and CB-Mon during Phase 3 (the final week of abstinence). Hypothesis 2.2 will be confirmed if abstinence is associated with lower (improved) affect/ideation ratings (component 1) and lower variance (component 2).

### Analysis for fitbit

We will conduct exploratory analyses of sleep, circadian rhythms, and physical activity. Sleep metrics from Fitbit include: *sleep duration*—mean and standard deviation (*SD*) over the main sleep period, daytime naps, and total 24-hour sleep; *sleep fragmentation*—mean and *SD* of 7-day sleep efficiency and wake after sleep onset; *sleep timing—*mean sleep onset, offset, and midpoint; *sleep timing variability—*across-day *SD* of sleep onset, offset, and midpoint. Physical activity metrics include*:—total daily activity counts and step count; Minutes in moderate-to-vigorous activity*. Circadian rhythm metrics will include: *relative activity-adjusted amplitude* [43] and *timing of peak activity (midpoint of M10)*. These variables will be summarized descriptively and examined in exploratory models of sleep–circadian–activity relationships.

### Missing data

Missing data in outcomes will be addressed automatically through maximum likelihood estimation of multilevel models. If there is missing covariate data (such as age and sex) and it does not exceed 5% of the sample size (participant-level), we will use single imputation. If missingness in participant-level covariates exceeds 5%, we will apply multiple imputation with predictive mean matching, pooling results over 48 imputations following Rubin’s rules [44].

### Data and safety monitoring

Data and safety monitoring will follow guidelines to protect human subjects overseen by the Mass General Brigham (MGB) institutional review board (IRB). All study procedures will be reviewed and approved by the MGB IRB.

This study will be overseen by the principal investigator, Dr. Randi Schuster, using a Data and Safety Monitoring Plan (DSMP). PI Schuster will hold regular meetings with all study investigators to review data collection, analysis details, and address any minor issues. Adverse events will be evaluated for each participant at every visit, except for the baseline visit. Unanticipated problems—defined as unexpected events that are related or possibly related to the research and indicate new or increased risks to participants—will be reported by phone or email to the MGB IRB following their current Adverse Event Reporting Policy.

An independent Data and Safety Monitoring Board (DSMB) has been appointed for this study to evaluate the safety of the clinical trial by determining whether there is an unacceptable level of risk due to cannabis abstinence, and whether an increased number of adverse events occur in the CB-Abst group compared with the CB-Mon group. The DSMB will comprise 4 members with expertise in (1) digital monitoring via smartphones, (2) longitudinal research with high-risk suicidal participants, (3) adolescents, and (4) biostatistics. Safety data will be reviewed by the DSMB every 6 months after the recruitment phase begins. The DSMB will receive summary reports on recruitment, retention, and all adverse events, which they will review at each biannual DSMB meeting. Subject information provided to the board will be identified only with study IDs to protect participant confidentiality.

### Ethics and dissemination

The protocol for this study has been carefully aligned with the most recent expert guidelines for ethical and safe research practices in conducting digital monitoring studies with individuals at risk for suicide. This includes procedures for monitoring and responding to incoming information about suicidal ideation from suicidal or self-injuring participants during the study [45,21]. Clinicians will be available during all study visits and EMA survey periods. Any significant increases in depressive symptoms [14] or suicidal ideation [29] will be recorded as adverse events and closely monitored until resolution.

The proposed study received IRB approval (#2024P002417; S6 file), the approved protocol is registered on ClinicalTrials.gov (NCT06576076), and upon completion of the study, the results will be reported to and made available on that site. All consent and assent forms will include a specific statement regarding the posting of clinical trial information on ClinicalTrials.gov. Any protocol changes will be submitted to the IRB for review and approval. Once approved, the modifications will be communicated to investigators and trial participants as needed to ensure transparency and compliance with reporting standards.

The study investigators are committed to the open and timely sharing of research results. All members of the research team agree to follow the NIH Grant Policy on Sharing of Unique Research Resources, including the Principles and Guidelines for Recipients of NIH Research Grants and Contracts on Obtaining and Disseminating Biomedical Research Resources issued on December 23, 1999.

### Confidentiality

Confidentiality will be preserved by assigning numerical codes to all data and storing it in password-protected databases. This study will also maintain a Certificate of Confidentiality from the National Institutes of Health. Some participants might worry that taking part in this protocol could reveal their substance use, especially to school staff and/or parents. During the telephone screening, if a participant is found eligible and chooses to enroll, we will ensure they understand that no information shared with study staff will be disclosed unless there are urgent safety concerns. We have implemented several safeguards, including masked language in the consent form, to lessen the chance that a participant’s parents or guardians will figure out that their child uses cannabis or shows symptoms of depression.

### Study status and timeline

The study status is ongoing. Participant recruitment and data collection began on January 1^st^, 2025. The expected end date for recruitment is July 30^th^, 2029 and the expected date for database completion is October 30^th^, 2029.

## Discussion

It is a public health priority to understand how cannabis intoxication and withdrawal affect depression and suicidal ideation in real-time, and to identify time-varying factors that may increase or decrease these effects. The proposed EMA protocol, used with a high-risk group, will help us clarify the links between cannabis use, depression, and suicidal thoughts. The knowledge gained will be essential in predicting when, why, and which individuals using cannabis experience changes in depression and suicidal ideation. This information will help identify modifiable targets for timely interventions during high-risk periods and support the development of effective, scalable real-time interventions for individuals at high risk of depression and/or suicide.

## Supporting information

S1 File Completed SPIRIT checklist. (DOCX)

S2 File Abstinence contract. (DOCX)

S3 File Abstinence planning sheet. (DOCX)

S4 File Ecological momentary assessment question bank. (DOCX)

S5 File Safety plan sheet. (DOCX)

S6 File Detailed protocol approved by the MGB IRB. (DOCX)
