# Supplementary material for: Characterizing proximal risk for depressive symptoms and suicidal ideation with acute cannabis use and withdrawal among adolescents using ecological momentary assessment: Study protocol
Source: PLoS One. 2025 Dec 18;20(12):e0338790. doi: 10.1371/journal.pone.0338790 (PMC12714289; doi:10.1371/journal.pone.0338790)
Supplement: S2 File — (DOCX) [file pone.0338790.s002.docx]

**Marijuana Abstinence Contract**

From Visit 2, __________(insert start date) to Visit 12, __________(insert end date), I agree that I will refrain completely from using marijuana. This includes using marijuana in any form (e.g., smoking, ingesting). After Visit 12, it will be up to me whether or not I resume using marijuana.

I understand that I will be drug tested at study visits 2-12. The drug tests used are very sensitive, so if the test does not show decreasing “marijuana levels,” then study staff may deem me to be non-abstinent regardless of whether I tell them that I have not used marijuana.

If I do not stop using marijuana completely for the full 56 days, then I may be asked to no longer participate in the study. Additionally, I understand that how much money I earn will depend on how many visits I attend and my ability to not use marijuana. If I attend every visit and do not use marijuana at all between Visits 2 and 12, I can earn $445.

- Attendance (Total: $445 if I come to all 12 Visits): $10 for visits 1 and 2, $5 each visit after visit 1 form those randomized to the abstinence group and $20 (+ 5 for each visit subsequent visit) for the monitoring group.

- Marijuana Abstinence (Total: $375 if I do not use marijuana at all between Visits 2-12): $15 for not using marijuana between Visits 2-3, $20 for not using between Visits 2-4, $25 for not using between Visits 2-5, $30 for not using between Visits 2-6, $35 for not using between Visits 2-7, $40 for not using between Visits 2-8, $45 for not using between Visits 2-9, $50 for not using between Visits 2-10, $55 for not using between Visits 2-11, and $60 for not using between Visits 2-12.

I will be compensated for attendance at the end of visits, and I will be compensated for abstinence between visits once my drug result tests come back from the laboratory. Someone from the study research staff will contact me to confirm how much money was loaded onto my card.

____________________ (Participant Signature)

____________________ (Researcher Signature)
