# Supplementary material for: Characterizing proximal risk for depressive symptoms and suicidal ideation with acute cannabis use and withdrawal among adolescents using ecological momentary assessment: Study protocol
Source: PLoS One. 2025 Dec 18;20(12):e0338790. doi: 10.1371/journal.pone.0338790 (PMC12714289; doi:10.1371/journal.pone.0338790)
Supplement: S3 File — (DOCX) [file pone.0338790.s003.docx]

Abstinence Plan

A guide to help you refrain from using marijuana throughout the study

**Step 1: Plans for the money**

You can earn up to $445 just for being abstinent! What would you use that for? Why is it important to you?

**Step 2: Getting started**

Get rid of any marijuana products/ paraphernalia. What do you have to get rid of and what will you do with it?

**Step 3: Plan for high-risk periods**

What are some potential triggers? Are there certain people with or places where you usually use marijuana?

**Step 4: Coping with negative mood**

What are some methods you can think of besides using marijuana to help deal with negative mood? (e.g. walking outside, journaling, watching TV)

**Step 5: Managing triggers**

Are there ways you can avoid your triggers? What can you say to people who might pressure you to use? What are some methods you might use to deal urges/cravings? (e.g. Urge Surfing + TIPP)

**Step 6: Support Systems**

Are there people in your life you can chat with when you are feeling like you want to use? Is there anything that we can do to help you stay abstinent? (e.g. texts check ins, providing motivational support, etc.)
