# Supplementary material for: Characterizing proximal risk for depressive symptoms and suicidal ideation with acute cannabis use and withdrawal among adolescents using ecological momentary assessment: Study protocol
Source: PLoS One. 2025 Dec 18;20(12):e0338790. doi: 10.1371/journal.pone.0338790 (PMC12714289; doi:10.1371/journal.pone.0338790)
Supplement: S4 File — (DOCX) [file pone.0338790.s004.docx]

**Question Bank for Smartphone Studies**

## **Questions asked at the first assessment of the day**

**What time did you try to go to sleep last night?**

- [Time]

**How well did you sleep last night?**

- [1 – 5 – 10] || **Very poorly – Fine – very well**

**How many hours did you sleep last night? _______**

**How much do you believe this statement:** “Today will be a good day.”

- [1% - 50% -100%] || **Not at all - Maybe – Completely**

**Substance Use Questions**

**Questions asked at each assessment**

When was the **most recent** time you used marijuana?

1. Within the last 0-15 minutes
2. Within the last 16-30 minutes
3. Within the last 31-60 minutes
4. I have not used in the last 60 minutes

- ***If anything besides “I have not used in the last 60 minutes”****:*
- How high did you get?
- [1 – 100 VAS] || **Not at all high – Extremely high**
- Did you use marijuana to improve your mood (e.g., to feel less depressed)?
  - [1 – 100 VAS] || **Not at All** – **Very Much**
    - ***If VAS > 1****:*
      - Was marijuana successful in improving your mood?
        - [1 – 100 VAS] || **Not at All** – **Very Much**
      - ***If “I have not used in the last 60 minutes”:***
- How much did you crave marijuana in the last 60 minutes?
  - [1 – 100 VAS] || **Not at All** – **Very Much**
- Have you used marijuana since the last time you completed a survey?
  - [No/Yes]
  - ***If yes****:*
    - How many times have you used marijuana since your last survey? [Integer only field]
    - When was the most recent date you used? If you used marijuana today, please put today’s date. [date fixed field]
    - When was the most recent time you used? [time fixed field]

**Affect Questions**

**Right now, how much do you feel:** [1 – 100 VAS] **|| Not at All - Very Much**

| **Affect Label** | **Definition (to be displayed along with label, but in a smaller font)** |
| --- | --- |
| Sad | Unhappy, down in the dumps, miserable |
| Isolated from others | N/A |
| Agitated | You are crawling out of your skin and cannot sit still |
| Happy |  |
| Irritated | You are easily bothered or annoyed by people or things around you |
| Anxious | Feeling like bad things could happen or are about to happen |
| Self-hatred | You hate who you are as a person |
| Excited | You feel energetic or eager |
| Numb | You have difficulty feeling emotions |
| Relaxed | You feel free from tension or anxiety |
| Hopeless | When things are bad you feel like things will never get better |
| Fatigued | You feel extremely tired or exhausted |
| Enthusiastic | You feel a lot of excitement or interest |
| Disinterested | You don’t want to do the things you usually find fun or interesting |
| Inattentive | You have difficulty focusing or paying attention |

***Attention Check****:* **Please move the slider to between 30- 40**

- [1 – 100]

**Suicidal Ideation Questions**

**In the past hour did you think about killing yourself?**

- [No/Yes]

**Right now, how strong is/ are your:** [1 – 100 VAS] || **Not at All** – **Very Strong**

- Thoughts about death
- Wish your suffering could all be over
- Thoughts you would be better off as dead
- Urge to kill yourself
- Urge to hurt your body (without wanting to die)

**Right now, how strong is your intention to kill yourself *today*?** [1 – 100 VAS] || **I am definitely not going to kill myself – I am definitely going to kill myself today**

*If response is 50 -79:*

| **We want you to stay safe. In a moment, we’ll show you some strategies and help that may be useful to you, including the safety plan you made at the beginning of the study.** |
| --- |
| **Please keep in mind, responses are NOT monitored in real time.** |
| **We strongly recommend that you call a therapist, call the suicide hotline (988), call 911, or go to the closest emergency room if you need any additional help, if your mood worsens, or if your thoughts of suicide get any worse.** |
| **On the next screen you’ll see the safety plan you made at the beginning of the study in case it’s helpful. Is there anything else you’d like to share with us about how you’re feeling right now?**  [open ended] |

*If response is 80 or above:*

| **We want you to stay safe. We’ll ask you two more questions and then we’ll show you the safety plan you made at the beginning of the study with strategies and help to keep you safe.** |
| --- |
| **Please keep in mind, responses are NOT monitored in real time.** |
| **Are you going to kill yourself today?**  [Yes, No, Maybe, Don’t Know] |
| **We strongly recommend that you call a therapist, call 911, call the Suicide Hotline (988) or go to the closest emergency room. Will you (check all that apply):**  [Call a therapist, Call 911, call the suicide hotline (988), Go to the emergency room, None of these] |
| **On the next screen you’ll see the safety plan you made at the beginning of the study in case it’s helpful. We will also check back in with you in approximately 15 minutes to see how you’re feeling. Is there anything else you’d like to share with us about how you’re feeling right now?**  [open ended] |

## **Safety Re-prompt**

*Comes up 15 minutes after a response of 80 or above on question: Right now, how strong is your intention to kill yourself today?*

**A little while ago, you indicated a strong intention to kill yourself today. Please respond to the following questions based on your feelings over the past 15 minutes to now:**

**The feelings I’m having right now are:**

- Not at all unbearable
- Mildly unbearable
- Somewhat unbearable
- Quite unbearable
- Extremely unbearable

**Right now, how strong is/are your** [1 – 100] || Not at all – Very strong

- Thoughts about death
- Wish your suffering could all be over
- Thoughts you would be better off as dead
- Urge to kill yourself
- Urge to hurt your body (without wanting to die)

**Right now, how strong is your intention to kill yourself *today*?** [1 – 100] || **I am definitely not going to kill myself – I am definitely going to kill myself today**

**Did you use any of the following resources?**

- Called a therapist
- Called 911 or emergency services
- Went to the emergency room
- Used a relax/distract skill
- Called a family member or friend
- None of the above

**Context/Connectedness Questions**

**Where are you right now?**

- At home
- At school
- At work
- Somewhere else
  - Please specify where you are right now:

**Right before you started this survey, who were you with?**

- Alone
- With people in person
- With people online/over the phone
- **If not alone:**
  - **Choose below who you were with.** (Check all that apply)
    - Friend - Romantic Partner
    - Acquaintance - Sibling
    - Parent/Step-parent - Teacher/ Instructor/ Coach
    - Classmate/coworker/colleague - Peer (not friend or acquaintance)
    - Alone - Other Family Member (e.g. Aunt)
    - Other - Children not in your family
    - Children in our family (e.g. niece, nephew, cousins, etc.)
      - Please specify the other people (not listed on the prior screen) who you were with right before you started this survey:
  - **How close or connected do you feel with these people right now?** [1 – 100 VAS] **|| Not close at all – Very close**

**Right before you started this survey, what were you doing?** (Check all that apply)

- Watching TV/ listening to music/ playing videogames
- Working (job or school work)
- Exercising/ playing sports
- Eating
- Resting or sleeping
- Socializing/ hanging out
- Texting, using social media, or browsing the web
- Doing a hobby (e.g., making art, doing yoga, etc.)
- Video Chat (Google Hangout, Skype, FaceTime)
- Housework/ chores
- Self-care (e.g. showering, grooming, etc.)
- In transit (e.g. driving, in car, on subway, etc.)
- Other
  - Please specify the other things you were doing (not listed on the prior screen) right before you started this survey:

## **Questions asked at the last assessment of the day**

**The next few questions are asking about the entire day today.**

**How much stress did you feel today?** [1 – 100 VAS] || **None – Very much**

**Did any of the following happen today? (**Check all that apply)

- Suicidal thoughts
- Made a suicide plan
- Attempted suicide
- Physically hurt yourself on purpose without trying to kill yourself

Drank alcohol

- - ***If checked:***
    - How many drinks did you have? (One drink is a can of beer, a glass of wine, a shot of liquor, or a mixed drink.): ______

Used marijuana

- - ***If checked:***
    - How many times did you use? ______
- Used other drugs (not including alcohol or marijuana)
  - ***If checked:***
    - **What other drugs did you use (not including alcohol or marijuana)?**_______
- Spoke to (or saw) a therapist
- Took mental health medication prescribed to you
- None

*The following questions are looped for each item endorsed in prior (check all) question*

**When did this event happen? Please try to be as accurate as possible, within one hour is best.**

*for suicidal thoughts & drugs, specify first and last time*

[Time and Date selector]
