# Supplementary material for: Characterizing proximal risk for depressive symptoms and suicidal ideation with acute cannabis use and withdrawal among adolescents using ecological momentary assessment: Study protocol
Source: PLoS One. 2025 Dec 18;20(12):e0338790. doi: 10.1371/journal.pone.0338790 (PMC12714289; doi:10.1371/journal.pone.0338790)
Supplement: S5 File — (DOCX) [file pone.0338790.s005.docx]

Safety Plan

A list of steps to keep you safe if you feel like hurting yourself.

**Step 1:**

**Relax/Distract**

(Examples: shower, listen to music, read a book or magazine, exercise)

**Step 2:**

**Talk to People**

(Examples: Call friends, family or therapist)

**Step 3:**

**Get Outside/ Additional/Formal Help**

(Example: go to local emergency room, call 988, call 911)
