# Supplementary material for: Characterizing proximal risk for depressive symptoms and suicidal ideation with acute cannabis use and withdrawal among adolescents using ecological momentary assessment: Study protocol
Source: PLoS One. 2025 Dec 18;20(12):e0338790. doi: 10.1371/journal.pone.0338790 (PMC12714289; doi:10.1371/journal.pone.0338790)
Supplement: S6 File — (DOCX) [file pone.0338790.s006.docx]

**Institutional Review Board**

**Intervention/Interaction Detailed Protocol**

Principal Investigator: Randi M. Schuster PhD

Project Title: Characterizing Proximal Risk for Depressive Symptoms and Suicidal Ideation with Acute Cannabis Use and Withdrawal Among Adolescents Using Ecological Momentary Assessment

Version Date: 10/15/2025

1. **Background and Significance**

**Historical background**

Adolescence is the period of highest risk for the initiation and escalation of cannabis use as well as the emergence of co-morbid psychopathology, including depression and suicidal ideation (SI). Nearly 1 in 2 U.S. adolescents have tried cannabis by 12th grade, with 1 in 15 using daily or near daily (Miech et al., 2019). Adolescent depression and suicide have also surged in recent years (Curtin et al., 2016). Past year major depression, SI, and suicide attempts occur in 1 in 10 (Bodden et al., 2018), 1 in 6 (Kann et al., 2018), and 1 in 14 adolescents (Kann et al., 2018), respectively. Cross-sectional and prospective studies find cannabis use co-occurs with depression and SI at alarmingly high rates (Gobbi et al., 2019). However, these distal associations do not reveal how cannabis use impacts intensity and variability in depressive symptoms and SI over shorter, clinically relevant periods of time (e.g., days, weeks, months), including intoxication and withdrawal.

Major depressive disorder and suicidal thoughts and behavior (STBs) are debilitating conditions associated with morbidity, mortality, and disability among adolescents. Teen depression and STBs may also be increasing as a result of COVID-19 (Kamenetz, 2020), and thus are likely to become an even greater public health crisis in the future. Adolescents between the ages of 15 - 24 years have the highest rates of mood disorders and SI (Kessler et al., 2005; Nock et al., 2008). Depression in adolescence is related to serious negative outcomes, including worse school performance and attendance, substance use, and SI (Glied & Pine, 2002; Fergusson & Woodward, 2002). These negative consequences persist across the life course; adults who were depressed in adolescence compared to those with adult onset depression have higher rates of adverse mental health outcomes, reliance on welfare, unemployment (Fergusson et al., 2007), and marital dissatisfaction (APA, 1998). The burden of depression is significant, as depression is among the leading causes of disability in both the United States and the world, and rates continue to increase (Reddy, 2010; James et al., 2018; NIMH, 2020).

Suicide is the second leading cause of death for adolescents ages 10-24 years (Curtin & Heron, 2019), accounting for more than 1 in 10 deaths (Kann et al., 2018; WHO, 2019), and rates have been increasing in the past decade (Curtin & Heron, 2019; Heron, 2019). SI is among the strongest predictors of subsequent suicide (Miranda et al., 2014; Wenzel et al., 2011; Mars et al., 2019). In adolescents, SI is also related to mental health disorders, poor family relationships, and poor school performance (Slap et al., 1989). STBs in adolescence result in an increased risk of adult SI and suicide attempts (Fergusson et al., 2005), suicide completion (Spirito et al., 1989), mental health disorders, unemployment, and long-term welfare use (Eckhoff et al., 2020; Briere et al., 2015).

**Previous pre-clinical or clinical studies leading up to, and supporting the proposed research**

Cross-sectional studies report associations between cannabis use and higher odds of depression (ORs: 1.2 - 1.7) (Degenhardt et al., 2013; Poulin et al., 2005; Rasic et al., 2013; Rey et al., 2002) and SI (ORs:1.3 to 2.5) (Carvalho et al., 2019; Huas et al., 2008; Sellers et al., 2019; Swahn et al, 2012), and longitudinal studies show elevated rates of subsequent depression and SI in young cannabis users even after adjustment for baseline covariates (Fergusson et al., 2002; Brook et al., 2002; Patton et al., 2002; Manrique-Garcia et al., 2012; Danielsson et al., 2016). A 30-year study found adolescent cannabis use to be associated with later depression and SI, but not anxiety, and these associations were strongest among those with early onset and high frequency adolescent use (Hengartner et al., 2020). These relationships have been substantiated in a recent meta-analysis (k=13) finding that adolescent cannabis use reliably predicts increased odds of development of depression (OR = 1.4), SI (OR = 1.5) and suicide attempts (OR = 3.5), but not anxiety (OR = 1.2), in young adulthood (Gobbi et al., 2019). Importantly, twin studies suggest that co-morbidity is unlikely to be fully attributable to predisposing genetic and/or environmental factors. Lynskey and colleagues (2004) found that cannabis dependent twins had nearly 3 times higher odds of SI than non-cannabis dependent co-twins, and odds were magnified when use was initiated before the age of 17. Similarly, Agrawal and colleagues (2017) found that, even after adjusting for confounds, monozygotic twins with frequent cannabis use were more likely to have depression (OR=2.0) and SI (OR=2.5) than monozygotic twins without frequent use.

The directionality of relationships between cannabis use and depression and SI among adolescents has received considerable attention. Premorbid psychological problems may increase risk for cannabis use as an attempt to ameliorate distress; (Bovasso, 2001; Bolanis et al., 2020) yet, more likely, cannabis may be a component in the multifactorial etiology of depression and SI. Gobbi and colleagues estimated the rate of young adult cases of depression likely attributable to cannabis exposure is ~7% (Gobbi et al., 2019). Further, recent genetic studies applying 2-sample Mendelian randomization have demonstrated potential causal contributions of cannabis use to SI and suicide attempts. These studies have demonstrated that there is a likely direct pathway from cannabis use, but not use of other substances, to suicide phenotypes, without evidence for reverse causation (Orri et al., 2020; Lim et al., 2020). **This provides strong preliminary evidence that cannabis use is an early, modifiable risk factor for depression and STBs.**

**There are plausible biological and socio-cultural mechanisms linking cannabis use, particularly during adolescence, to phenotypic risk for depression and SI.** The brain undergoes substantial maturation throughout adolescence involving critical synaptic and structural changes in circuits, such as the mesocorticolimbic pathway, that mediate cognition, self-regulation, and emotional processing (Giedd et al., 1999; Fuhrmann et all., 2015). Introduction of exogenous substances during this sensitive period may interfere with these ongoing neurodevelopmental processes. Heavy and early use of cannabis may cause insult and increase risk for mood problems through neurochemically altering effects of the main biologically active phytocannabinoid, ∆-9-tetrahydrocannabinol (THC), on the endocannabinoid system, which plays an important role in modulating mood in both animal (Hill & Gorzalka, 2009) and human models (Agrawal et al., 2012). Regular cannabis use decreases the availability of CB1 receptors in areas of the brain related to mood disorders (Hirvonen et al., 2012), and genetic or pharmacological blockade of CB1 receptors produces a behavioral syndrome similar to depression in animals (Gorzalka & Hill, 2011). Further, preclinical studies have shown exposure to cannabinoids in adolescence but not during adulthood results in aberrant monoaminergic neurotransmission, including decreases in serotonin and increases in norepinephrine, as well as behavioral increases in anhedonia (Bambico et al., 2010; Pistis et al., 2004; Rubino & Parolaro, 2016). In addition, cannabis use increases exposure to environmental risk factors and social determinants, such as trauma, other substance use, affiliation with deviant peers, and poor educational attainment (Werner et al., 2016; Volkow et al., 2014; Silins et al., 2014) that, in turn, increase likelihood of developing depression and SI (APA, 1994; Kandel et al., 1991). **Together, this cumulatively suggests that regular cannabis use, despite sometimes used to cope with depressive symptoms (Azcarate et al., 2020), may actually make users more susceptible to the effects of depression through direct and indirect pathways.**

**Rationale behind the proposed research, and potential benefits to patients and/or society**

**While there is convincing evidence that cannabis use is a distal risk factor for adverse mental health outcomes, research on the effects of acute cannabis use on proximal risk for depression and SI, particularly among high-risk adolescents, is sparse.** It is not known whether depression and SI reflect a direct, pharmacogenic consequence of adolescent cannabis use, or whether this co-morbidity is better accounted for by common risk factors (e.g., environmental, genetic, personality, biological factors).

To our knowledge, acute effects of cannabis on mood have not been evaluated among chronic adolescent cannabis users and those with co-morbid depression and SI. This represents a vulnerable sub-group due to the combination of risk factors conferred by ongoing neurodevelopmental vulnerability, early and heavy use, and psychiatric co-morbidity. This high-risk group may also report using cannabis to cope with distress (Bottorff et al., 2009), which is robust predictor of greater cannabis-related problems and increased depressive symptoms (Bonn-Miller et al., 2014; Moitra et al., 2015). **It is critical that we discover how cannabis use acutely impacts mood and SI in depressed adolescent users to elucidate whether, when and for whom use modulates risk for adverse mood and suicide outcomes and to define the factors that may maintain use (e.g., temporary relief of distress) in this high-risk population.** We aim to leverage advances in mobile device-based data collection to uncover time-varying, momentary relationships between acute cannabis use and depression and SI among adolescents.

1. **Specific Aims and Objectives**

To make an impact on rising rates of depression and suicide in adolescents, we must improve our ability to predict *when*, *why, and which* individuals experience increases in depression and SI. **This study seeks to disentangle relationships between acute cannabis use and withdrawal on proximal depression and suicide risk and recovery by incorporating time-varying patterns of substance use, mood, and SI, and guide the development of scalable, individualized, accessible, and affordable interventions aimed to reduce depression and suicide risk among adolescents (N=200).**

**Aim 1:** To test if negative mood and SI precipitate cannabis use events and whether negative mood and

SI are temporarily relieved and stabilized through acute cannabis use (i.e., negative reinforcement).

*Hypothesis 1.1:* Negative mood will predict subsequent same-day cannabis use events as well as greater self-reported motivation to use cannabis for mood improvement (EMA Phase 1; Baseline as Usual).

*Hypothesis 1.2:* Negative mood and SI reported within one hour following cannabis use will be lower in intensity and less variable than mood and SI reported during same-day random-prompt EMA reports occurring ≥1 hours since last cannabis use (EMA Phase 1; Baseline as Usual).

*Hypothesis 1.3 (exploratory):* There will be within-subject correlates (e.g., concurrent other substance use, social context of use) and between-subject factors (e.g., cannabis use and depression severity, history of suicidal behavior) that moderate relationships between acute cannabis use and mood and SI.

**Aim 2:** To test if negative mood and SI decrease in overall (mean) levels and variability after cannabis

wash-out.

*Hypothesis 2.1:* Among CB-Abst but not CB-Mon, negative mood and SI reported during random prompt EMA reports will intensify and become more variable throughout the first week of sustained cannabis withdrawal (EMA Phase 2; Randomized Withdrawal) compared to random, non-use events during baseline cannabis use as usual (EMA Phase 1; Baseline as Usual).

*Hypothesis 2.2:* Negative mood and SI reported during EMA random prompts after 8 weeks of abstinence (EMA Phase 3; Randomized Sustained Abstinence) will be lower in intensity and variability than negative mood and SI reported during EMA random prompts occurring during random, non-use events during baseline cannabis use as usual (EMA Phase 1; Baseline as Usual) and withdrawal (EMA phase 2; Randomized Withdrawal). There will be no difference in mood or SI intensity or variability across the 3 EMA phases in CB-Mon.

1. **General Description of Study Design**

We will conduct a 10-week, multi-method, randomized study, and will recruit 200 school-based adolescents, ages 12-18 years, with daily or near daily cannabis use and current depressive symptoms. The study design is nearly identical to that used in another IRB approved protocol aimed at pilot data collection for the current protocol (#2021P002416), apart from minor modifications to the study population. Participants will complete 2 weeks of real-time EMA during baseline cannabis use (EMA Phase 1; Baseline Use as Usual) to quantify the temporal relationship between cannabis use and mood and SI, and the within- (e.g., concurrent other substance use, social context of use) and between-subject factors (e.g., average severity of cannabis use, depression and SI) that may moderate these linkages. To determine how depression and SI change across short- and longer-term cannabis withdrawal, participants will then be randomized to 8 weeks of monitoring (CB-Mon) or incentivized abstinence via contingency management (CB-Abst). A key aspect of the approach is that the 8-week period is long enough to observe true changes due to cannabis discontinuation, separate from effects of withdrawal and residual cannabinoid exposure that generally subside in 4 weeks. Both groups will complete 1 week of EMA in weeks 1 (EMA Phase 2; Randomized Withdrawal) and 8 (EMA Phase 3; Randomized Sustained Abstinence) of the randomized study period. During all 3 EMA phases, participants will receive 7-9 random, time-stamped prompts per day to ascertain mood and SI in the absence of acute cannabis use, proximal but prior to cannabis use, and during or shortly after cannabis use.

**Figure 1: Study Arms Overview**

Enrollment Visit

Randomization Visit

EMA (1 week; Phase 3)

**Time**

**Visit Number**

**Study Week**

**Abstinence**

**Monitoring**

Baseline Cannabis Use

EMA (2 Weeks; Phase 1)

EMA (1 week; Phase 2)

1 2 3 4 5 6 7 8 9 10 11 12

0 1 2 3 4 5 6 7 8 9 10

5 – 15 min visit

30 – 40 min visit

90 – 180 min visit

**Key**

*****

**= pt. will have flexibility to allow scheduling period**

*****

**A**

**= abstinence begins**

**A**

1. **Subject Selection**

Eligibility criteria are identical to those in IRB approved pilot protocol #2021P002416, apart from the upper age limit.

**Inclusion Criteria**

1. Ages 12-18;
2. Current daily or near daily cannabis use (i.e., use ≥ 4 days per week on average; Timeline Followback);
3. Score ≥ 5 on PHQ-9;
4. Access to an internet-capable smartphone (iOS or Android);
5. Provision of at least 1 collateral contact for risk monitoring;
6. Provision of informed assent (or consent if 18 years) and parent/guardian consent if <age 18;
7. Approximately 50% response rate to EMA prompts during the first EMA phase (Baseline as Usual);
8. No immediate plan to discontinue cannabis use in the next 3 months;
9. Positive toxicology result for cannabis on baseline urinalysis.

**Exclusion Criteria**

1. Any factor that impairs ability to comprehend and effectively participate, including acute intoxication at time of consent;
2. Cannabis use >4 times/day on average (to maximize likelihood of capturing mood and SI during non-use times);
3. Inability to speak/write English fluently;
4. Gross cognitive impairment, for example due to florid psychosis, intellectual disability, developmental delay, or neurodegenerative disease;
5. Current epilepsy diagnosis;
6. Individuals who are under the legal protection of the government or state (wards of the state);
7. Response of “No” to the knowledge check question regarding EMA suicidality response time.

So as to not exclude individuals refusing care and/or who cannot access care due to barriers, we are not requiring participants to have a current treater to be considered eligible. However, we will liaise with parents if there is a safety issue and will also require an additional emergency contact if we cannot reach parents. A collateral contact may also be contacted in the instance study staff is unable to contact a participant after mutliple failed contact attempts. Once eligibility is confirmed, the baseline visit will be scheduled, at which time written parental consent and student assent will be obtained.

**Local Recruitment Procedures**

All recruitment procedures are identical to those used in IRB approved pilot protocol #2021P002416. Participants will be adolescents between the ages of 12-18 years and referred to the study via: 1) annual on-campus or virtual screenings as part of a separate IRB-approved screening protocol (#2021P001873), 2) through an IRB-approved universal phone screening protocol (#2024P002895) designed to systematically assess eligibility for multiple IRB-approved studies at once, and 3) on a case by case basis through direct physician, teacher, administrator referrals and parent or participant request. Participants will be referred through the community via postings online, flyers, and through social media advertisements (Twitter, Reddit, Snapchat). We will also work with a recruiting platform, BuildClinical, to advertise on social media platforms. All advertisements will be IRB approved. Potential participants will complete a telephone screen for eligibility. We are will use OutFront, an MGB-approved vendor, to expand our study outreach for recruitment. Study flyers will appear across a network of MBTA trains and busses that link to the study MGB Rally page. In addition, participants will be recruited using the Research Patient Data Registry (RPDR) through MGB, a clinical data registry that can identify patients for clinical trials. We will run queries on RPDR to find subjects with current depression, current suicidal ideation, and current cannabis use reported for at least >4 days per week, therefore likely to meet our eligibility criteria for this research study. Subjects identified through these mechanisms and who have not opted out of receiving research invitations will receive a personalized letter through their patient gateway account detailing parts of the study and contact information for our study staff, should they be interested in learning more. For those subjects who are under the age of 18, research initiations will be sent to parents/legal guardians that have not opted out of receiving such letters.

1. **Subject Enrollment**

**On-Site Screening and Enrollment**

A brief overview of the school screening procedures from our other IRB approved school recruitment protocol (#2021P001873), which will serve as a recruitment pipeline for the current study, is outlined below. Screening will begin with a school-wide assessment to characterize the demographics and general behavioral profile of the schools attended by the participants, and to identify participants most likely to be eligible for this study. Participants will be recruited by MGH study staff and not by any district and/or school personnel. Recruitment will occur through an opt-out procedure. The school-wide assessment will be distributed several days after contact is made to all parents/guardians to ensure that they have adequate time to discuss questions with MGH study staff and/or withdraw their children from the school-wide assessment should that be their preference. During the time of the school-wide assessment, MGH study staff will distribute a brief questionnaire to all students who were not opted-out by their parents/guardians. This questionnaire will include general demographic items as well as basic questions related to health behavior and psychological functioning. All students who are not withdrawn from the school-wide assessment by their parents/guardians and who wish to complete the questionnaire will also complete a separate form where they may provide their contact information and indicate their interest in being contacted for research studies. The contact information form and the brief questionnaire will be separated to protect individual confidentiality. Questionnaires will then be reviewed only by MGH study staff, and all students who indicate a willingness to be contacted will be considered potentially eligible, pending eligibility confirmation via telephone screen and receipt of written parental consent (see below). The procedures of the intervention component of the study will not begin for any participant (under the age of 18) until the parental consent form is obtained.

**Procedures for Obtaining Informed Consent**

**Consent for Screening—Waived Parental Consent for participants under age 18:** Given the history of low response rate of studies requiring students to return parent permission forms (which limits the number of students who can be involved in the project and undermines the generalizability of the results, thus limiting the usefulness of our research findings), initial screening will occur in part through a separate IRB school recruitment protocol (#2021P001873), through an IRB-approved universal phone screening protocol (#2024P002895), or directly at local, participating schools with passive parental consent for screening. However, active parent consent (for those <18 years of age) and participant assent will be required to enroll in the intervention component of the study.

The opt-out survey procedure for initial screening currently underway is detailed completely in the existing school recruitment protocol (#2021P001873). Briefly, participating schools will contact all parents/guardians of 6^th^ through 12^th^ grade students using the school’s preferred method (e.g., through reverse 9-11 calls, emails, mass mailings) indicating that MGH study staff will be distributing and collecting a very brief questionnaire during a designated time at school. Parents and guardians will be told that they can freely withdraw their child from participating in the school-wide assessment without any penalty or consequence to the child, including any impact on school or educational activities, and that parental written consent must be provided to MGH staff for participation in any additional research activities (including the intervention study proposed here). Parents and legal guardians will be informed that this is not a study or program of the respective school district, including a program of the specific middle or high school. School personnel will not be involved in administrating or operating the study in any capacity, including answering student and/or parent or legal guardian inquiries regarding the study. Parents and legal guardians will be free to contact the principal investigator of this study at MGH directly at any time if they have questions about the school-wide assessment or the specific items included in the questionnaire. Parents can deny passive consent for the school-wide assessment by contacting MGH study staff during a designated window of time.

Likely eligible and interested participants will be contacted directly after completion of the school-wide assessment to confirm eligibility. Self-referred participants, or those referred by parents or other school or mental health professionals will similarly be contacted by phone to assess eligibility.

We request a waiver of parental consent for those under age 18 for *phone screening only,* which is justified given the following:

- **The proposed phone screening involves no more than minimal risk to participants.** During screening, we will provide details about the study and ask potential participants if they have any questions or concerns about the study protocol. We will also ask participants basic demographic information as well as brief questions related to symptoms of psychopathology to assess eligibility. These questions are indistinguishable from questions asked during standard school mental health screening assessments (e.g., SBIRT). This screener is anticipated to take no more than five minutes and participants are free to refuse to answer any questions. They will be told that all information will be kept strictly confidential and answers to screening questions will be stored separately from any identifying information. If participants are interested in the study and are eligible, we will collect parent/guardian contact information so that we can obtain written parental consent for study enrollment. No study procedures will occur before written parental consent is obtained. We will also obtain written adolescent assent at the baseline visit, prior to the initiation of any study procedures, during which time we will review the study in detail.
- **The waiver of parental consent for phone screening will not adversely affect the rights and welfare of subjects.** The waiver of parental consent for screening only will not adversely impact an adolescent’s access to or the nature of any ongoing health services or primary care in any way nor adversely affect his/her rights or welfare. The study will be detailed to potential participants before any screening questions are asked of them, giving them a chance to decline screening if they are not interested in the study. They are also free to refuse to answer any screening questions that make them feel uncomfortable. They will be clearly told that written parental consent and adolescent assent will be required for enrollment in the active study phase and that answers to phone-based questions are for screening purposes only.
- **The research could not practicably be carried out with a waiver of parental consent for phone screening.** This study could not be practicably carried out without a waiver of parental consent for phone screening for eligibility confirmation due to the burden that would be imposed on parents of potential participants that are not yet even known to be eligible and interested in study participation. This study utilizes a brief, de-identified telephone screen to assess whether the potential participant is interested and eligible for the study. Screening questions are similar to questions asked in standard school assessments. In our experience, the consent process generally takes approximately 30 minutes, while the brief screener should only take about 5-10 minutes for most participants. Requiring consent for screening would thus dramatically increase parent burden, and in many cases, this imposed burden would be unnecessary because it is not guaranteed that their child is interested or eligible for the study. Due to this increased parent burden, we expect that requiring consent for screening would greatly reduce enrollment and compromise the representativeness of the sample.
- **Participants and parents will be provided with additional pertinent information after participation.** Prior to the first study visit, written parental consent will be obtained (for subjects under age 18) and parents will be provided with copies of the consent form which will fully detail the study protocol as well as any associated risks and benefits. They will be given contact information of key study staff if they would like more information. Similarly, study participants will be given a copy of the signed parental consent/study assent form.
- **The phone screening involves no more than minimal risk to the privacy of the participants.** This research involves no more than minimal risk to the privacy of participants. During the screening, only basic demographics and information to determine eligibility will be collected. The protocol fully details our plan to protect any identifying information from improper use and disclosure.

**Adolescents Recruited and Enrolled Under 18 Years of Age: Written Parental Consent and Student Assent:** Parent/guardian contact information will be collected from potential participants who expressed interest in participating in the active study phase, who were found to be eligible on the telephone screen, and who are under the age of 18. After a participant is determined to be eligible, study staff will reach out to the parent/guardian (for participants under the age of 18) to schedule a time to perform consent procedures in person or via telephone or virtual visit (via MGB-approved video conference platforms like Microsoft Teams or zoom) with a licensed clinician on study staff. Should we find that a student speaks a language spoken by a member of our study staff, but their parent/guardian does not, we will employ the MGB-approved short-form consent method to obtain parent/guardian consent before seeking participant consent. Participants will similarly perform consent/assent procedures with a licensed clinician on study staff at the baseline study visit. A doctoral level clinician, including PI Dr. Randi Schuster (licensed psychologist), will be on call during all consenting and assenting procedures. The participant will be informed over the phone and/or via email that the signed parental consent form must be received from his/her parent/guardian prior to, or at, the first visit. All participants will be given the opportunity to ask questions to a member of the study staff during the consent/assent process. Contact information of key MGH study staff will be provided and participants (and parents/guardians) will be informed that the co-investigators are available to answer any questions or concerns they may have about the study. All participants as well as their parents/guardians will be provided with a copy of their signed parental consent and adolescent assent forms. No study procedures will begin until parental consent is obtained (for participants under the age of 18). Minors who turn 18 while enrolled in the study will be re-consented using the adult consent form to continue participation.

Parents/guardians have the option to sign the consent form in person, or electronically via Adobe System. Adobe System’s digital signature feature is a secure and commonly used method of obtaining signatures electronically. Parents/guardians may reach out to MGH study staff with any questions or concerns at any time prior to or after signing the consent form.

**Adolescents Recruited and Enrolled who are 18 Years of Age:** Potential participants who are interested in participating and are 18 years old will work directly with MGH staff following completion of the telephone screen to coordinate the initial study visit. At the time of such visit, informed consent will be obtained by a licensed clinician on study staff. Consent will be obtained for the potential participant via telephone, virtual visit (via MGB-approved video conference platforms like Microsoft Teams or zoom), or in-person. All participants will be given the opportunity to ask questions to a member of the study staff during the consent process. Contact information of key study staff will be provided, and participants will be informed that the co-investigators are available to answer any questions or concerns they may have about the study. All eligible participants will be provided with a copy of their signed consent form.

Parents/guardians and participants will be told that participation is voluntary, and that if they choose not to participate, their status at the participating school will not be affected. All participants as well as their parents/guardians will be provided with a copy of their signed parental consent and student assent forms.

**Treatment Assignment and/or Randomization**

If participants meet eligibility criteria and a signed consent form has been received from a parent/guardian, they may be contacted and scheduled for a baseline session. For participants aged 18 years, baseline sessions may be scheduled prior to obtaining consent (as consent will be obtained at baseline prior to administering any study procedures). For all participants, a member of the study team will discuss the consent/assent form with the participant, provide answers to any questions, and, if the participant is interested, a licensed clinician will obtain informed consent/assent. The participant will then be given a signed copy of the consent/assent form.

Once enrolled and after the baseline phase (EMA Phase 1; Baseline as Usual), participants will be scheduled for randomization, which will employ a 2-arm, within-subject, parallel group design to examine effects of short- and longer-term withdrawal across 8 weeks of cannabis abstinence on depression and SI. Participants will be randomized to 8 weeks of abstinence with contingency management (CB-Abst: n=100) or non-contingent monitoring with no abstinence requirement (CB-Mon: n=100) using a 1:1 ratio in blocks of 6. A subset of study staff who will administer surveys and questionnaires querying about mood and suicidality will be blind to group assignment. Randomization will be based on a computerized scheme developed by the study statistician. Randomization will be stratified by age (<=14 years old vs >=15 years old), sex (male vs female), and depression severity (mild/moderate vs. Moderately severe/severe). A key aspect of the approach is that the 8-week period is long enough to observe true changes due to cannabis discontinuation, separate from effects of cannabis withdrawal and residual cannabinoid exposure. Groups will complete 1-week of EMA in weeks 1 (EMA Phase 2; Randomized Withdrawal) and 8 (EMA Phase 3; Randomized Sustained Abstinence) of the randomized study period.

Individuals randomized to monitoring who incidentally stop using cannabis, or express an interest in discontinuing cannabis, will not be removed from the study. If desired, these individuals will be provided with substance use resources at the time of interest in cessation.

1. **STUDY PROCEDURES**

Study procedures are nearly identical to those used in IRB approved pilot protocol (#2021P002416).

**Screening (V0):** School-wide screening will take place on site and at the participating schools (as part of #2021P001873), and/or over the phone. Data will be collected during the screening to determine eligibility for the study.

*Measures to be completed by participants:*

- School-wide screening instrument (as part of #2021P001873; only for participants recruited through schools)
- Telephone screener
- Universal screener (as part of #2024P002895)

**Baseline Visit (V1):** Participants will attend an approximately 3-hour baseline visit to be conducted on the campus of the participating school, at a local library, at Massachusetts General Hospital, or virtually via video conferencing (via MGB-approved video conference platforms like Microsoft Teams or zoom). The baseline visit will initiate a 2 week-long baseline phase involving smartphone EMA during which they will use cannabis per usual to quantify the temporal relationship between use and mood and SI, and the within- (e.g., concurrent other substance use, social contexts) and between-subject factors (e.g., severity of cannabis use, depression, and SI) that moderate these linkages. We will ensure that this 2-week baseline phase does not coincide with when there are anticipated major deviations from typical patterns of cannabis use (e.g., family vacation, exam week).

*Eligibility confirmation to be completed prior to obtaining consent:*

Patient Health Questionnaire-9 (PHQ-9)

*Measures to be completed by participants following consent:*

- Timeline Followback Interview (TLFB)
- Substance Use History
- Marijuana Craving Questionnaire – Short form (MCQ-SF)
- Cannabis Withdrawal Scale (CWS)
- Marijuana Motives Measure (MMM)
- Drug Testing
- Ecological Momentary Assessment (EMA) Training
- The Reasons of Living for Adolescents (RFL-A)
- Self-injurious Thoughts and Behaviors Interview-Revised (SITBI-R)
- Child Behavior Checklist Youth Self Report (CBCL)
- Columbia Suicide Severity Rating Scale (C-SSRS)
- Pittsburgh Sleep Quality Index (PSQI)
- Mini International Neuropsychiatric Interview (MINI) Kid 7.0.2: Cannabis Use Disorder and Major Depressive Episode
- Beck Anxiety Inventory (BAI)
- Alcohol Use Disorder Identification Test (AUDIT)
- The Cannabis Use Disorder Identification Test (CUDIT)
- Penn State Electronic Cigarette Dependence (ECDI)
- Connors ADHD Self-Report Index (ASRI)
- Psychiatric History
- Concomitant Medications
- Demographics
- *Fitbit Set-up

* For virtual participants, Fitbits will be sent immediately following the baseline visit and set-up asynchronously

**Visits 2-12 (V2-V12)**

- **Visit 2 (V2):** Visit 2 (V2) will take approximately 45 minutes to complete. At V2, participants will be randomized to 8 weeks of abstinence with contingency management (CB-Abst: n=100) or non-contingent monitoring with no abstinence requirement (CB-Mon: n=100).

*Measures to be completed by participants:*

- Timeline Followback Interview (TLFB)
- Marijuana Craving Questionnaire – Short Form (MCQ-SF)
- Cannabis Withdrawal Scale (CWS)
- Drug Testing
- The Reasons of Living for Adolescents (RFL-A)
- Columbia Suicide Severity Rating Scale (C-SSRS)
- Patient Health Questionnaire-9 (PHQ-9)
- Pittsburgh Sleep Quality Index (PSQI)
- Family Psychiatric History (KSADS)
- Short UPPS-P Impulsive Behavior Scale (Short UPPS-P)
- Monetary Choice Questionnaire Delayed Reward Discounting (MCQ Delay Discounting)
- Adverse Events
- Concomitant Medications
- Social Network and Substance Use
- **Visit 3 – 4 (V3-V4):** Visits 3 – 4 will take approximately 15 – 45 minutes

*Measures to be completed by participants:*

- Timeline Followback Interview (TLFB)
- Marijuana Craving Questionnaire – Short Form (MCQ-SF)
- Cannabis Withdrawal Scale (CWS)
- Drug Testing
- Columbia Suicide Severity Rating Scale (C-SSRS)
- Patient Health Questionnaire-9 (PHQ-9)
- Adverse Events
- Concomitant Medications
- **Visit 5 – 11 (V5-V11):** Visit 5 – 11 will take approximately 45 minutes

*Measures to be completed by participants:*

- Timeline Followback Interview (TLFB)
- Marijuana Craving Questionnaire – Short Form (MCQ-SF)
- Cannabis Withdrawal Scale (CWS)
- Drug Testing
- The Reasons of Living for Adolescents (RFL-A)
- Columbia Suicide Severity Rating Scale (C-SSRS)
- Patient Health Questionnaire-9 (PHQ-9)
- Pittsburgh Sleep Quality Index (PSQI)
- Adverse Events
- Concomitant Medications
- **Visit 12 (V12):** Visit 12 will take approximately 45 minutes

*Measures to be completed by participants:*

- Timeline Followback Interview (TLFB)
- Marijuana Craving Questionnaire – Short Form (MCQ-SF)
- Cannabis Withdrawal Scale (CWS)
- Beck Anxiety Inventory (BAI)
- Drug Testing
- Connors ADHD Self-Report Index (ASRI)
- The Reasons of Living for Adolescents (RFL-A)
- Columbia Suicide Severity Rating Scale (C-SSRS)
- Patient Health Questionnaire-9 (PHQ-9)
- Pittsburgh Sleep Quality Index (PSQI)
- Adverse Events
- Concomitant Medications
- Social Network and Substance Use
- ***Exit Interview:** Phone call will take approximately 10 minutes.

*Measures to be completed by participants:*

- PHQ-9
- Exit interview questions

*this is an optional interview that will only occur for participants who express that they do not want to complete all study procedures

- At visit 12 all participants, regardless of group assignment, will be provided treatment and support resources.

**Assessment Techniques**

Participants will complete 12 study visits across ~10 weeks and three waves of EMA data collection. To minimize burden and maximize feasibility, study visits will be conducted in a private room at a participant’s school campus or local library, in lab settings, or virtually via video conferencing (via MGB-approved video conference platforms like Microsoft Teams or zoom). Visits will entail collection of urine samples for verification of self-reported use and abstinence verification (for CB-Abst post-randomization), as well as brief questionnaires and interviews on interim substance use, craving, and withdrawal. Questionnaires and surveys on mood symptoms and suicidal ideation (SI) will be conducted by study staff blinded to group assignment.

**Semi-Structured Interviews:** Substance use disorders and other psychiatric diagnoses will be queried at the baseline with past and current modules of the Mini International Neuropsychiatric Interview (MINI) Kid 7.0.2, which was designed as a brief structured diagnostic interview for the major psychiatric disorders across development in DSM-5 (Sheehan, Sheehan, & Shytle et al., 2010). We will evaluate for cannabis use disorder, major depressive disorder, as well as other Axis I diagnoses (including those specific to developmental populations). At the baseline visit, we will administer the Self-Injurious Thoughts and Behaviors Interview (Nock et al., 2007) to assess lifetime self-injurious thoughts and behaviors and confirm past month SI. We will also conduct the Columbia-Suicide Severity Rating Scale (Posner et al., 2011) at V1 to capture in-depth information about past month SI. At V2-12, SI and behavior since last visit will be determined with the Columbia-Suicide Severity Rating Scale *(alongside the primary corresponding EMA measure).* Interviews will be administered by trained study staff with established interrater reliability.

Participants will complete a timeline follow-back (TLFB) (Sobell et al., 1986) for frequency, quantity, and duration of nicotine, cannabis, alcohol, and any other recreational substance use. A 90-day recall period will be queried at the baseline visit, and a past week recall period will be queried at subsequent study visits; V2-12). Based on our decades-worth of experience conducting adolescent substance use research, we do not anticipate adolescents being unwilling to report on such activities despite substance use being illegal for underage persons. Our proposed protocol will use best practices for maximizing validity of substance use self-report, including guaranteeing confidentiality, utilizing interviews designed to aid recall via memory aids, training interviewers to standardize interviewing techniques and give instruction on developing rapport with the participant and probing incongruent answers, emphasizing the lack of consequences for accurately reporting substance use, and explaining the existence of external, objective validation of substance use via drug tests (Babor et al., 1987). Cannabis and other substance use is not highly stigmatized among adolescents (Roditis & Halpern-Felsher, 2015); therefore, the likelihood of underreporting is expected to be very low. Additionally, and as is our practice in our ongoing trials, participant self-reported substance use will be treated as strictly confidential and will not be disclosed to parents at any time (Gryczynski et al., 2019), except in cases of suspected serious harm to self or others (e.g., driving while intoxicated). This will be explicitly explained to parents and participants during the consent/assent process.

**Questionnaires:** Surveys will be administered via REDCap, a HIPAA compliant, web-based application hosted by Partners HealthCare designed to support data capture for research studies (http://rc.partners.org/edcredcap), at in-person visits (or virtually via MGB-approved video conference platforms like Microsoft Teams or zoom; V1-12). Construct areas queried include, for example: demographics; cannabis use of family cannabis withdrawal; current and past use of other substance of abuse (e.g., tobacco, alcohol); family rules about substance use; parental messages about substance use; quality of family communication; peer support; social integration; exposure to risky peer networks; self-esteem; coping; depressive, anxiety, and ADHD symptoms; delinquent behaviors; temperament; suicidal ideation; sleeping habits. To enhance reproducibility, measures were drawn from the PhenX Toolkit when available (Hamilton et al., 2011).

**Toxicology:** At all study visits, participants will provide a urine sample, which will be used to qualitatively screen for cannabis use as well as other substances of misuse and quantitatively screen for amount of THC metabolites in urine.

For visits conducted virtually (via MGB-approved video conference platforms like Microsoft Teams or zoom), participants will be given or mailed a qualitative testing and shipping kit. Participants will be guided through the steps of testing the urine sample collected at each visit and will be instructed on how to package the sample for shipment.

**Ecological Momentary Assessment (conducted between V1-V2, V2-V5 and V11-V12):** At baseline, participants will be guided through installing the EMA platform (MetricWire) on their iOS or Android smartphone. Support for app installation and EMA training will be provided throughout the study as needed by phone and via tutorial materials. Participants will be told that surveys will commence the next day and that they should use the app throughout the next 2 weeks in the same way as trained at baseline. During the EMA phases, participants will complete random prompts via MetricWire using a data sampling scheme similar to that used in prior studies (Shiffman et al., 2020). Participants will never be asked to complete >9 random EMA surveys/day. To enhance compliance, participants will be paid a $100 compliance bonus at the end of each EMA phase if ≥80% of random prompts are completed.

At the end of 2 weeks, the app will automatically discontinue EMA surveys. Two 1-week EMA phases will occur during the randomized period (weeks 1 and 8 post randomization; Figure 1). Staff will re-train participants on the EMA app prior to each of these EMA phases. Staff members will monitor EMA compliance and will reach out proactively to participants failing to meet criteria. After the third and final EMA phase, participants will be reminded that they will no longer need the EMA app, and that they can delete it (see figure 1 study schema for illustration of EMA administration periods).

All EMA surveys, regardless of phase of the study, will consist of short, multiple-choice items with logic-branched follow-up items. Within each of these surveys, participants will be queried on social settings and activities, cannabis and other substance use, mood, and suicidal ideation. See sections below detailing our plan for data monitoring to ensure safety in the event of SI exacerbation.

**Actigraphy Component (Fitbit): P**articipants will participate in a study component that collects sleep and activity data via a Fitbit Inspire 3. Participants will wear the watch after enrollment at Visit 1 through the Visit 12 (approximately 70 days after enrollment). To enhance compliance, participants will be paid a $25 compliance bonus at each of the three EMA phases data is available for 16/24 hours of 80% of days within that phase.

| **Passive Parental Consent** | **Parental/student consent/assent** |  | **Cannabis users in CM discontinue cannabis use** |  |  |  |  |  |  |  |  |  |  |  |  |
| --- | --- | --- | --- | --- | --- | --- | --- | --- | --- | --- | --- | --- | --- | --- | --- |
|  | **V0** |  | **BL (V1)** | **V2** | **V3** | **V4** | **V5** | **V6** | **V7** | **V8** | **V9** | **V10** | **V11** | **V12** | **Exit interview** |
| Days since Baseline |  |  | 0 | 14 | 16 | 19 | 21 | 28 | 35 | 42 | 49 | 56 | 63 | 70 |  |
| Fitbit Inspire 3 |  |  | x | x | x | x | x | x | x | x | x | x | x | x |  |
| Phone Screener | x |  |  |  |  |  |  |  |  |  |  |  |  |  |  |
| EMA Assessments  (x indicates when EMA assessment phases begin) |  |  | x | x |  |  |  |  |  |  |  |  | x |  |  |
| *Substance Use Questionnaires* |  |  |  |  |  |  |  |  |  |  |  |  |  |  |  |
| AUDIT |  |  | x |  |  |  |  |  |  |  |  |  |  |  |  |
| CUDIT |  |  | x |  |  |  |  |  |  |  |  |  |  |  |  |
| CWS |  |  | x | x | x | x | x | x | x | x | x | x | x | x |  |
| ECDI |  |  | x |  |  |  |  |  |  |  |  |  |  |  |  |
| MCQ-SF |  |  | x | x | x | x | x | x | x | x | x | x | x | x |  |
| MMM |  |  | x |  |  |  |  |  |  |  |  |  |  |  |  |
| TLFB |  |  | x | x | x | x | x | x | x | x | x | x | x | x |  |
| Substance Use History |  |  | x |  |  |  |  |  |  |  |  |  |  |  |  |
| *Suicidal Ideation and Behavior* |  |  |  |  |  |  |  |  |  |  |  |  |  |  |  |
| BAI |  |  | x |  |  |  |  |  |  |  |  |  |  | x |  |
| PHQ-9 |  |  | x | x | x | x | x | x | x | x | x | x | x | x | x |
| Columbia Suicide-Severity Rating Scale |  |  | x | x | x | x | x | x | x | x | x | x | x | x |  |
| RFL-A |  |  | x | x |  |  | x | x | x | x | x | x | x | x |  |
| SITBI-R |  |  | x |  |  |  |  |  |  |  |  |  |  |  |  |
| *Other Relevant Confounds and Covariates* |  |  |  |  |  |  |  |  |  |  |  |  |  |  |  |
| ASRI |  |  | x |  |  |  |  |  |  |  |  |  |  | x |  |
| Adverse Events |  |  |  | x | x | x | x | x | x | x | x | x | x | x |  |
| Concomitant Medications |  |  | x | x | x | x | x | x | x | x | x | x | x | x |  |
| Demographics |  |  | x |  |  |  |  |  |  |  |  |  |  |  |  |
| Family Psychiatric History (KSADS) |  |  |  | x |  |  |  |  |  |  |  |  |  |  |  |
| PSQI |  |  | x | x |  |  | x | x | x | x | x | x | x | x |  |
| MCQ Delay Discounting |  |  |  | x |  |  |  |  |  |  |  |  |  |  |  |
| Medical Psychiatric History |  |  | x |  |  |  |  |  |  |  |  |  |  |  |  |
| Child Behavior Checklist Youth Self Report (CBCL) |  |  | x |  |  |  |  |  |  |  |  |  |  |  |  |
| MINI (MDE and CUD) |  |  | x |  |  |  |  |  |  |  |  |  |  |  |  |
| UPPS-PS |  |  |  | x |  |  |  |  |  |  |  |  |  |  |  |
| Social Network and Substance Use |  |  |  | x |  |  |  |  |  |  |  |  |  | x |  |
| *Urinalysis Test* |  |  |  |  |  |  |  |  |  |  |  |  |  |  |  |
| Toxicology |  |  | x | x | x | x | x | x | x | x | x | x | x | x |  |
| *Exit Interview* |  |  |  |  |  |  |  |  |  |  |  |  |  |  | x |

**Platforms for Data Collection**

**Questionnaires Collected via Research Electronic Data Capture (REDCap):** Surveys will be administered via REDCap, a HIPAA compliant, web-based application hosted by Partners HealthCare designed to support data capture for research studies (http://rc.partners.org/edcredcap), at in-person visits (or virtually via MGB-approved video conference platforms like Microsoft Teams or zoom; V1-12).

Data will be stored automatically and securely on a SQL Server, accessed over industry standard TLS 256 bit RSA encryption during data transfers. Data is routinely backed up locally onto a redundancy server and stored in a separate database. Long term storage on Partners servers occurs nightly and allows for incremental backup over multiple systems. Therefore, should one drive be physically damaged, there will be multiples within the chain to replace it. Both data servers are stored within PHS IS corporate firewall, in a secure, key access facility with password-protected computers. Only vetted PHS security officials will have access to physical machines storing study data. Since data are stored on a protected server, a compromise of any individual computer at a research facility will not lead to a breach of the secure database. Individual computers designated for data capture do not store participants’ identifying information or study data.

**MetricWire (iOS & Android OS):** (<https://metricwire.com/>): EMA data capture will occur through Metricwire**,** a secure smartphone-based application with full-cycle project capabilities, including and most importantly for the current project is in-the-moment observation and reporting. This application is easily customizable to build sophisticated protocols using a simple drag-and-drop interface to engage participants under virtually any scenario and capture contextually relevant data. The application can be programmed with more the 60 different types of question formats, has both mobile and web participant-facing applications, and has functionality to notify participants of surveys to-be-completed via push notifications, SMS, voice, and email. Compliance management workflows deliver actionable information to ensure participant safety, maximize response rates and keep the study team on the same page. Relevant to this high-risk cohort, individualized safety plans can be pre-programmed into the application and can be delivered electronically to participants if safety concerns are reported during any EMA prompt type. Metricwire is currently the only EMA platform available that complies with HIPAA, FDA 21 part 11 and GDPR. All data collected will be stored with a unique random ID, making identification from survey responses impossible.

Aim 1: Primary outcomes will be depression items (e.g., bored, sad, tired, irritable) and SI intensity items (presence, urge, controllability) which are measured on a scale of “1 – Not at all” to “100 – Very Much” after each EMA prompt during the phase 1 of EMA.

Aim 2: Primary outcomes will be depression items (e.g., bored, sad, tired, irritable) and SI intensity items (presence, urge, controllability) which are measured on a scale of “1 – Not at all” to “100 – Very Much” after each EMA prompt during phases 1, 2 and 3 of EMA.

**Fitbit Protocol:** The purpose of the Fitbit portion of the study is to assess whether cannabis abstinence impacts objective measures of sleep in school-aged adolescents and young adults. At Baseline, participants will receive a Fitbit 3 that is paired with a premade, de-identified account. At the first visit, participants will be instructed on how to use the device, including charging and syncing. Participants will then be instructed to wear the device at all times (except when charging the device), from Baseline (Visit 1) through Visit 12 (approximately 70 days after Baseline). Participants will be instructed to sync their device frequently throughout the study. At each visit, a member of study staff will also sync the Fitbit to the Fitbit app to pair the deidentified sleep and activity data to the pre-made de-identified account.

**Remuneration**

Participants may be paid between study visits for their participation. The level of compensation is deemed appropriate given the time commitment. Reloadable cards will be managed by Advarra Participant Payments. Advarra Participant Payments is an MGH-approved, secure, web-based platform that facilitates clinical trial and study-related payments onto prepaid reloadable Visa cards. Participants will be informed of Advarra policy during the consent process. After agreeing to participate in the study, participants will also be asked to sign a “Advarra Participant Payment Card Acknowledgement of Receipt” form to certify their understanding of Advarra Participant Payment policies. The form will require participants to print and sign their name and provide their social security number. If the participant is under 18 years old, then parent/guardian information including date of birth, social security number, and address will be collected. Advarra acknowledgment forms will be stored in a locked study cabinet. Given that the scientific objective of this study necessitates implementation of a robust contingency management protocol which requires immediate payment following verification of cannabis abstinence, the primary payment delivery method will be Advarra. If a participant is under the age of 18 and does not have a parent that is willing to provide a social security number, they may be paid via MGB-issued check. Social security numbers will be stored and protected on the MGB-approved, secure REDCap server. Participants will be compensated for attendance of in-person visits, abstinence (if randomized to CB-Abst), plus a compliance bonus at the end of each EMA phase if ≥80% of random prompts are completed. Cannabis abstinence will be indexed by self-reported non-use and, given cannabis’ long half-life, by progressively decreasing quantitative levels of 11-nor-delta-9-THC-9-carboxylic acid (THCCOOH), the primary cannabis metabolite, in urine. Laboratory processing of cannabis metabolites will be conducted at Dominion Diagnostics.

Payments will be made using an escalating scale for abstinence reimbursements and on a consistent scale for EMA compliance (see Tables 1 & 2). The reimbursement schedule was informed by PI’s ongoing contingency management studies and has been effective in other populations (Worden et al., 2017). Maximum total earnings will be matched across group to not confound the potential effects of monetary payments and depressive symptoms and SI. Attendance payments paid via Advarra will be issued at the end of every in-person visit (when participants provide urine samples), abstinence payments will be made between visits when abstinence is biochemically confirmed, and EMA payments will be issued at the end of each of the three EMA phases.

| **Table 2: Max Overall Study Remuneration Schedule** | |
| --- | --- |
| **EMA Compliance Phase 1 (approximately 80%) completion** | $100 |
| **Or: EMA Compliance Phase 1 (approximately 50%) completion** | $25 |
| **Fitbit Compliance Phase 1 (66%) completion** | $25 |
| **EMA Compliance Phase 2 (approximately 80%) completion** | $100 |
| **Or: EMA Compliance Phase 2 (approximately 50%) completion** | $25 |
| **Fitbit Compliance Phase 2 (66%) completion** | $25 |
| **EMA Compliance Phase 3 (approximately 80%) completion** | $100 |
| **Or: EMA Compliance Phase 3 (approximately 50%) completion** | $25 |
| **Fitbit Compliance Phase 3 (66%) completion** | $25 |
| **Study Visits/ Abstinence** | $445 |
| **Overall Fitbit and EMA Bonus (completion across whole study)** | $50 |
| **Max Total Earnings $870** | |

Participants will earn either $100 for a compliance rate of approximately 80% or $25 for a compliance rate of approximately 50% across each of the three EMA phases and $25 for a Fitbit compliance rate of 66% across each of the three phases. Participants can earn an additional $50 if they achieve an overall compliance rate for both Fitbits and EMA phases across the entire study. Payments will be made at the study completion. If a Fitbit device is lost or not returned, 20% of the total earned for this portion of the study will be deducted.

**Text Message Procedures**

Participants may receive text messages throughout their time in the study. Text messages will be sent either via Televox or Google Voice. Any messages containing sensitive information, such as patient health information, will be sent using Televox, as it is HIPAA compliant and secure. No messages will be sent without first receiving participant consent. Consent to receive text messages will be documented in the participant’s study file. Text messages may include communications about recruitment, scheduling, visit reminders, survey compliance updates, abstinence confirmation, and safety check ins.

**Termination of Participation**

Participants will be terminated from this study if they fail to comply with study procedures or if in the opinion of the principal investigator, can no longer safely participate.

1. **Risks and Discomforts**

**Potential risks in the study are considered minimal and include:** Psychosocial, Social and Legal Risks: There are no uncommon psychological, social, or legal risks associated with participation in this study. Periods of voluntary cannabis abstinence are not uncommon for adolescent users as they often take breaks for participation in sports, family vacations, etc. There is a slight risk that the questionnaires and interviews may contribute to temporary discomfort when participants are asked about distressing symptoms (e.g., suicidal thoughts). Notably, prior research has shown that answering questions about suicidal thoughts and actions does not increase psychological distress or increase one’s suicidal thoughts/behaviors (Reynolds et al., 2006). Nevertheless, a study staff member will monitor the EMA responses and in the event of significantly worsening depressive symptoms, suicidal thoughts, or behaviors (described in detail below), will alert a clinically trained staff member who will follow-up within 24 hours. A doctoral level clinician, including PI Dr. Randi Schuster, will be on call should they be needed.

**Fitbit Risks:** Fitbits used for sleep and activity data pose minimal risk and do not alter the current level of risk for the main study. A small portion of people either find the device uncomfortable or have reactions to the device or band. Alternate material Fitbit bands will be ordered as needed.

**Confidentiality and Privacy**: Another potential risk to participants is breach of confidentiality. However, protecting the confidentiality and integrity of our research participants is a top priority for this MGH project, and the risk of loss of privacy is judged to be minimal. Data will be collected using REDCap (Research Electronic Data Capture; http://rc.partners.org/edcredcap) tools (Harris 2009) hosted with Partners HealthCare. Assessments will be administered via REDCap and smartphone EMA. REDCap, REDCap Survey systems, and smartphone EMA provide secure, HIPAA compliant, web-based applications. Confidentiality will be maintained by numerically coding all data and by keeping all data in password protected databases. In addition, this study will maintain a Certificate of Confidentiality from the National Institutes of Health. Some participants may be concerned that participating in this protocol will disclose their substance-using habits, particularly to school personnel and/or parents. During the telephone screener, if a participant is determined eligible and would like to be enrolled, we will make sure to tell participants that no information shared with study staff will be disclosed unless there are imminent safety concerns. We have taken several precautions, including masking language in the consent form, to reduce the likelihood that a participant’s parents/guardians will deduce that their child uses cannabis and has symptoms of depression. However, it is impossible to remove all risk that parents/guardians may deduce that their child uses cannabis and has symptoms of depression because we are only recruiting cannabis users who report depressive symptoms. During the telephone screener, we will make sure participants are aware of this and give them the opportunity to decline participation before any contact is made with parents. Additionally, both participants and parents/guardians will be made aware that no details disclosed during the study, including those relevant to use or mental health or substance use history, will be shared with the parents/guardians unless there is an immediate concern for safety.

There is a potential that we may need to break confidentiality for the proposed study. During the informed consent process, we will inform participants that all information they provide as part of the study is confidential, however, there is a risk that we will break confidentiality if the participant is at serious risk of harm to self. Our plan for risk monitoring and breaking of confidentiality adheres to guidelines set forth by a previously approved IRB protocol (#2015P000598) and a recent consensus statement from experts in the field of suicide and EMA, carefully considering participant safety without introduction of potentially iatrogenic effects involving reactivity to contingent risk assessment in EMA (Nock et al., 2021). Specifically, if a participant indicates serious risk of suicide (i.e., if they rate their intention of acting on their suicidal thoughts as 80 or more out of 100 on the EMA prompt), the EMA program will automatically prompt the participant again 15 minutes later. If the second prompt is not completed or if symptoms to not dissipate below 80/100, then a safety check-in by a clinically trained staff member will be initiated within 24 hours either by text (Televox; a HIPPA complaint, MGB-approved texting platform) or phone call. If a safety check in is initiated with the participant, parents/guardians will also be notified. We will have dedicated study staff for twice daily monitoring of daily EMA data 7 days a week for risk determinations. Phone-based risk assessments will only be conducted by a clinically trained staff member with a doctoral level clinician on standby.

If a participant rates their intention of acting on their suicidal thoughts as a 50 out of 100 or higher, the smartphone EMA application will send a push notification to the participant’s smartphone stating that (1) app data is not monitored in real time and (2) the participant should contact a member of their treatment team, call the suicide hotline (1-800-273-8255), or go to the nearest emergency room if they need additional help. This will ensure that participants at elevated but not imminent risk will still have access to suicide risk reduction resources. At the baseline visit, we will personalize a safety plan that will be programmed into the EMA application and “pushed” to the participant if he/she rates his/her intention of acting on suicidal thoughts as a 50 out of 100 or higher.

Parents and participants will be made clear of our risk monitoring protocol and plans for breaking confidentiality at the point of consent and throughout the protocol as necessary.

1. **Benefits**

**Potential Benefits of the Proposed Research to Human Subjects and Others**

Participants may find that responding to questions about depressive symptoms and suicidal ideation increases their awareness of these experiences, or that monitoring makes them feel more connected to or cared for by the research staff, which would promote improved mental health. Additionally, as we will continually monitor risk for suicide throughout the study and contact participants for risk assessments as necessary, participants may be protected against risk of suicidal behavior. Finally, our experience with adolescents participating in similar research is that they often enjoy the opportunity to interact with field staff, to engage in an interesting self-study, and to feel as if they are making a contribution to research on the lives of other teenagers. There will be no cost to participants.

**Potential Benefits to Society**

Information developed from this study may help researchers in the future. The expected scientific yield of the project is substantial. There is much to be learned from the project. Findings have the potential to have measurable implications on policy, prevention, and treatment efforts. The greatest benefit will be to future cohorts of adolescents who will benefit from what is learned from this research project.

The risks associated with this study are minimal and reasonable, particularly in light of the fact that significant knowledge will be gained.

1. **Statistical Analysis**

**Data preparation.** Six negative affect measures will be defined via composite averages and individual scores using EMA items: 1) the average over 5 items assessing depleted mood (scores for feeling Sad, Self-hatred, Numb, Hopeless, and Fatigued), 2) the average over 2 items assessing negative cognitive impacts (scores for feeling Disinterested and Inattentive), 3) the average over 3 items assessing negative activation (scores for feeling Agitated, Irritated, and Anxious), 4) the average over 3 items assessing passive suicidal ideation (scores for Thoughts about death, Wishing suffering could be over, and Better off as dead), 5) the individual item for suicidal urges, and 6) the individual item for non-suicidal self-injury urges. All items will be measured on a 0 (No, not at all) – 100 (Yes, very much) scale. Acute cannabis use will be defined as a binary indicator variable (1 = self-reported use in the hour preceding the current random prompt, 0 = no use in the last hour).Additionally, whether cannabis was used in the last hour to improve negative mood will be assessed on a 0 (No cannabis use) and 1 (No, not at all) – 100 (Yes, very much) scale.

**Statistical models.** Separate analyses will be conducted for each negative affect measure. Statistical models will assume that a) data are a mixture of a non-zero continuous component and zeros, and b) the continuous component will follow a beta distribution (i.e., scores will be scaled to be between 0 and 1). Models will include 1) a participant-varying lag-1 autocorrelation term (to handle repeated measures) and 2) a participant-varying probability term governing the occurrence of zeros (to allow for individual differences). Additional covariates will include weekday (categorical, Monday-Sunday), time of day (categorical, 6:00-11:00, 11:00-16:00, 16:00-21:00), age (continuous), and gender (categorical). To test specific hypotheses, additional predictors will be included as needed for a) the beta regression, b) the residual variance term of the beta regression, and c) the probability term governing zeros.

**Data analysis.**

**Aim 1: To test if negative mood and SI are temporarily relieved and stabilized through acute cannabis use (i.e., negative reinforcement cycle).** We will only use phase 1 data, the 2-week baseline period in which all participants may use cannabis. *Hypothesis 1.1:* We hypothesize that negative affect measures for a) the current random prompt, and b) the prior random prompt (a lag-1 term) will predict 1) greater cannabis use independent of motivating reason (i.e., lower probability for the zero component of the model), and 2) higher scores for motivated cannabis use to improve negative mood. Negative affect measures will therefore be converted to z-scores for this analysis and included as predictors for both the zero and non-zero components of the model. *Hypothesis 1.2:* We hypothesize that the binary indicator for cannabis use in the last hour will predict lower, less variable scores for the negative affect measures. Therefore, the binary use indicator will be included as a predictor for the beta regression residual variance term as well. *Hypothesis 1.3 (exploratory):* There will be within-subject correlates (e.g., concurrent other substance use, social context of use) and between-subject factors (e.g., cannabis use and depression severity, history of suicidal behavior) that moderate relationships between acute cannabis use and negative affect.

**Aim 2: To test if negative mood and SI initially increase with the start of abstinence and then decrease in both mean levels and variability after cannabis wash-out.** We will use phase 1 – 3 data, including predictors for a) group differences (CB-Abst vs. CB-Mon), b) differences by phase (phase 3 vs. phase 1 and phase 2 vs. phase 1), and c) terms for the group by phase interactions. All terms will be included as predictors for the beta regression residual variance term as well. We will only include data for the CB-Abst group with confirmed abstinence for phases 2 – 3. *Hypothesis 2.1:* We hypothesize that CB-Abst will show higher mean negative affect scores and greater variability during phase 2 compared to phase 1, while CB-Mon will show no differences between phases. *Hypothesis 2.2:* We hypothesize that CB-Abst will show lower mean negative affect scores and less variability during phase 3 compared to phase 1, while CB-Mon will show no differences between phases.

**Exploratory analyses:** To further assess heterogeneity in suicidal ideation, we will conduct a set of

exploratory analyses for hypotheses 1.1 – 2.2 looking at all 5 individual items for suicidal ideation. To examine the impact of participants who are versus are not receiving mental health treatment, we will conduct a set of exploratory analyses for hypothesis 1.1 – 2.2 including a binary indicator for receipt of mental health treatment as an additional covariate. Additionally, we will conduct exploratory analyses for sleep and circadian rhythms. Sleep, circadian, and physical activity exploratory Fitbit metrics include: *Sleep duration*: mean and standard deviation [StD]) over the main sleep period, the day, and 24hrs; short (<6 hrs) and long (>9hrs) mean nocturnal sleep duration; *Sleep fragmentation*: Mean and StD of 7-day sleep efficiency and wake after sleep onset; *Sleep timing placement*: Mean sleep onset, offset, and mid-point; categorized also as advanced (sleep onset <0900) and delayed (sleep onset >0200); *Sleep timing variability:* Across-day StD of sleep onset, inter-and intra-day stability; sleep offset, and sleep mid-point; relative activity- adjusted amplitude (Roberts et al., 2020); *Physical activity:* Overall activity, time spent walking, in moderate and physical activity. Timing of maximal physical activity will be used in analyses of rest-activity rhythm. Data will be processed in a customized data management environment that interfaces with Fitbit cloud servers. This platform provides secure data acquisition and management tools that facilitate remote data collection without the need to return devices for data extraction. Using an in-house algorithm that captures continuous heart rate sensor data at 1-5-sec intervals, we will calculate minutes of non-wear time (i.e., absence of a pulse for ≥60 secs), enabling us to determine compliance and distinguish between real and spurious sedentary time. Missing data will be handled using interpolation. Labeled sleep stage data will be retrieved at a granularity of 30s, activity counts at 60s, and the heart rate at 1s. Sleep-wake metrics will be generated using signals from accelerometry, heart rate, and heart rate variability processed by Fitbit’s proprietary, validated algorithm (Dillon et al., 2005). Valid sleep data will be defined as non-nap sleep durations >3 hr. Physical activity will be derived from daily step counts detected by Fitbit’s triaxial accelerometer, which agrees with direct observation, and will be reported as daily and intraday (per minute) step counts. Using step counts, we will calculate minutes spent doing sedentary, light, moderate, and vigorous activity. Metrics will be calculated for each 24-hour period, generating averages and within subject standard deviations (to assess within-subject variability).

**Multiple testing.** We will use the false recovery rate adjustment to control for multiple testing across the 6 negative affect measures separately for each hypothesis (Benjamini, 1995).

*Sleep & Circadian Rhythms (Exploratory).* Fitbits will be worn throughout the trial to assess changes in sleep. Sleep, circadian, and physical activity exploratory Fitbit metrics include: *Sleep duration*: mean and standard deviation [StD]) over the main sleep period, the day, and 24hrs; short (<6 hrs) and long (>9hrs) mean nocturnal sleep duration; *Sleep fragmentation*: Mean and StD of 7-day sleep efficiency and wake after sleep onset; *Sleep timing placement*: Mean sleep onset, offset, and mid-point; categorized also as advanced (sleep onset <0900) and delayed (sleep onset >0200); *Sleep timing variability:* Across-day StD of sleep onset, inter-and intra-day stability; sleep offset, and sleep mid-point; relative activity- adjusted amplitude;192 *Physical activity:* Overall activity, time spent walking, in moderate and physical activity. Timing of maximal physical activity will be used in analyses of rest-activity rhythm.

**Missing Data***.* Some data will inevitably be missing. We will explore patterns of missingness to determine if missingness is occurring at random (MAR) (i.e., unrelated to the value of the missing observation) or missing not at random (MNAR). For each analysis, we will use a variety of recommended strategies to address the issue of missing data (e.g., multiple imputation, maximum likelihood estimation) (Schafer & Graham, 2002). Consistency in findings across missing data methods will enhance our confidence in the findings. If findings are discrepant, we will assume that participants randomized to CB-Abst have resumed using cannabis, if they are lost to follow-up.

**Protocol non-adherence.** Analyses involving phase 1 and/or phase 2 of EMA data collection, will only consider CB-Abst participants who have biochemically-validated CB abstinence for two months because the goal is to study the effect of abstinence on change in mood and SI. If fewer than expected CB-Abst abstain from CB use for the full two months, we will form Helmert contrasts: those in CB-Mon (a), CB-Abst who do not abstain (b), and CB-Abst who abstain (c). The 1^st^ Helmert contrast will compare a to b+c and will be the intent to treat comparison. The 2^nd^ contrast will compare b to c, which corresponds to the post hoc sensitivity analysis of interest. Similar considerations will be made if participants in CB-Mon incidentally discontinue CB use during the study.

**Power analysis and justification of recruitment targets for the confirmatory cohort.** The R package ‘EMAtools’ (version 0.1.3; Kleiman, 2017) was used to determine the power for the proposed analyses. Preliminary analyses from the exploratory study found effect size estimates for significant effects ranged from 0.19 to 0.44, and that 77% of participants completed all phases of the study, completing on average 76% of all prompts. Estimates of the power were therefore computed to detect a weak effect (a Cohen’s d of .2) based on linear multi-level models using the nesting structure for the proposed EMA analyses and an intra-class correlation of .09. Table 1 shows the breakdown of estimates power based on the hypothesis being tested under two scenarios: 1) an ideal scenario with the full proposed number of participants and responses, and 2) a realistic scenario where 77% of participants complete 76% of entries.

Table 1: Power estimates by hypothesis and attrition scenario (ideal versus worse-case)

| Hypothesis | N (%) for sample size | N (%) of completed entries | Power |
| --- | --- | --- | --- |
| 1.1 - 1.2 | 200 (100%) | 112 (100%) | 99% |
|  | 154 (77%) | 85 (76%) | 93% |
| 2.1 - 2.2 | 200 (100%) | 224 (100%) | 99% |
|  | 154 (77%) | 170 (76%) | 94% |

1. **Monitoring and Quality Assurance**

**Reporting Adverse Events (AEs)**

PI Schuster will meet regularly with all study investigators to review the details of data acquisition and analysis as well as any minor problems. AEs will be assessed for each subject at every in-person visit. In the event of any minor or significant AE, the PI will be contacted immediately. The PI will be responsible for the generation of summary reports documenting this process and outcomes which will be included in the Continuing Review reports to the IRB. Serious AEs (events that are unexpected, and related or possibly related to the research and that indicate there are new or increased risks to subjects) will be reported by telephone or email to the Partners IRB according to current PHRC Adverse Event Reporting Policy. All AEs (if not serious) will be reported in writing to the Partners Human Research Committee via Insight. All information regarding experimental subjects will be kept in a locked drawer in PI Schuster’s locked office. All data will be identified by a unique code number.

**Planned Safety Monitoring**

This study will be monitored by PI, Dr. Randi Schuster, using a Data and Safety Monitoring Plan (DSMP). It is considered necessary for the purpose of monitoring the safety of the study that PI review not only AEs and SAEs, but other data that may reflect differences in safety between treatment groups. This includes treatment retention rates, reasons for drop-out, and clinical outcomes. The main purposes of the DSMP will be to (1) ensure the continued safety of study participants, (2) monitor enrollment and retention, (3) confirm that there are no issues with data confidentiality, and (4) determine whether there is an unacceptable level of risk and increase number of adverse events due to cannabis abstinence. The PI will have overall responsibility for safety and data monitoring on a day-to-day basis.

An independent Data and Safety Monitoring Board (DSMB) will be appointed for this study to assess safety of this clinical trial by determining whether there is an unacceptable level of risk due to cannabis abstinence and whether an increased number of adverse events occur in the CB-Abst group compared with the CB-Mon group. A DSMB will be created at the beginning of year 1, as an independent body charged with ensuring that the safety of study subjects is protected and that the scientific goals of the study are being met. The DSMB will consist of four members whose expertise spans fields such as (1) digital monitoring via smartphones, (2) longitudinal research with high-risk suicidal participants, (3) adolescents, and (4) biostatistics. Each member of the DSMB will not otherwise be associated with the trial and the study investigators will not be members of the DSMB. Safety data will be reviewed by the DSMB every 6 months after the recruitment period begins. The DSMB will receive summary reports on recruitment, retention and description of all adverse events and review them at each biannual DSMB meeting. Subject information provided to the board will be identified only with study IDs to protect the confidentiality of subjects.

**Outcomes Monitoring**

Quality assurance procedures will include training of all study personnel (including psychology/psychiatry trainees) prior to initiation of the study and monitoring of subject enrollment and ensuring quality of data (source documentation and online in RedCap). Internal audits will be conducted under the supervision of the principal investigator, to assure that study data in RedCap are accurate and in agreement with source documentation, verify that consent for the study has been properly obtained and documented, and confirm that participants enrolled in the study meet inclusion and exclusion criteria. Any significant deviations from the protocol, including non-adherence to inclusion/exclusion criteria, errors in study procedures, or failure to complete assessments, will be reported to the Partners HRC as a protocol violation.

Participants will be terminated from this study if they fail to comply with study procedures, or if the opinion of the principal investigator, can no longer safely participate.

**Internal Monitoring (source data, protocol adherence, recordkeeping)**

The principal investigator and co-investigators will meet regularly with all study investigators to review the details of data acquisition and analysis as well as any minor problems. All study personnel will be trained on the collection of source data, protocol adherence, and recordkeeping as required by the MGB Research Institute. Primary research assistants will review source data, protocol adherence, and records daily and meet with the principal investigator and co-investigators weekly. The primary research assistants will also be responsible for the maintenance of training records for new study personnel.

1. **Privacy and Confidentiality**

Study procedures will be conducted in a private setting

Only data and/or specimens necessary for the conduct of the study will be collected

Data collected (paper and/or electronic) will be maintained in a secure location with appropriate protections such as password protection, encryption, physical security measures (locked files/areas)

Specimens collected will be maintained in a secure location with appropriate protections (e.g. locked storage spaces, laboratory areas)

Data and specimens will only be shared with individuals who are members of the IRB-approved research team or approved for sharing as described in this IRB protocol

Data and/or specimens requiring transportation from one location or electronic space to another will be transported only in a secure manner (e.g. encrypted files, password protection, using chain-of-custody procedures, etc.)

All electronic communication with participants will comply with Mass General Brigham secure communication policies

Identifiers will be coded or removed as soon as feasible and access to files linking identifiers with coded data or specimens will be limited to the minimal necessary members of the research team required to conduct the research

All staff are trained on and will follow the Mass General Brigham policies and procedures for maintaining appropriate confidentiality of research data and specimens

The PI will ensure that all staff implement and follow any Research Information Service Office (RISO) requirements for this research

Additional privacy and/or confidentiality protections

PI will ensure that all staff are trained on proper consent procedures to ensure participants and parents of minors are aware of situations where confidentiality needs to be broken to ensure safety.

**12. References**

American Psychological Association. (1994). Adolescent psychopathology: II. Psychosocial risk factors for depression [press release].

American Psychological Association. (1998).Consequences of depression during adolescence: Marital status and marital functioning in early adulthood [press release].

Agrawal, A., Nelson, E. C., Bucholz, K. K., Tillman, R., Grucza, R. A., Statham, D. J., Madden, P. A., Martin, N. G., Heath, A. C., & Lynskey, M. T. (2017). Major depressive disorder, suicidal thoughts and behaviours, and cannabis involvement in discordant twins: A retrospective cohort study. *The Lancet. Psychiatry*, *4*(9), 706–714. <https://doi.org/10.1016/S2215-0366(17)30280-8>

Agrawal, A., Nelson, E. C., Littlefield, A. K., Bucholz, K. K., Degenhardt, L., Henders, A. K., Madden, P. A. F., Martin, N. G., Montgomery, G. W., Pergadia, M. L., Sher, K. J., Heath, A. C., & Lynskey, M. T. (2012). Cannabinoid receptor (CNR1) genotype moderates the effects of childhood physical abuse on anhedonia and depression. *Archives of General Psychiatry*, *69*(7), 732–740. <https://doi.org/10.1001/archgenpsychiatry.2011.2273>

Azcarate, P. M., Zhang, A. J., Keyhani, S., Steigerwald, S., Ishida, J. H., & Cohen, B. E. (2020). Medical Reasons for Marijuana Use, Forms of Use, and Patient Perception of Physician Attitudes Among the US Population. *Journal of General Internal Medicine*, *35*(7), 1979–1986. <https://doi.org/10.1007/s11606-020-05800-7>

Babor, T. F., Stephens, R. S., & Marlatt, G. A. (1987). Verbal report methods in clinical research on alcoholism: Response bias and its minimization. *Journal of Studies on Alcohol*, *48*(5), 410–424. <https://doi.org/10.15288/jsa.1987.48.410>

Bambico, F. R., Nguyen, N.-T., Katz, N., & Gobbi, G. (2010). Chronic exposure to cannabinoids during adolescence but not during adulthood impairs emotional behaviour and monoaminergic neurotransmission. *Neurobiology of Disease*, *37*(3), 641–655. <https://doi.org/10.1016/j.nbd.2009.11.020>

Baron KG, Duffecy J, Berendsen MA, Cheung Mason I, Lattie EG, Manalo NC. Feeling validated yet? A

scoping review of the use of consumer-targeted wearable and mobile technology to measure and improve sleep. Sleep Med Rev. Aug 2018;40:151-159. doi:10.1016/j.smrv.2017.12.002

Benjamini, Y., & Hochberg, Y. (1995). Controlling the False Discovery Rate: A Practical and Powerful Approach to Multiple Testing. *Journal of the Royal Statistical Society. Series B (Methodological)*, *57*(1), 289–300.

Bodden, D. H. M., Stikkelbroek, Y., & Dirksen, C. D. (2018). Societal burden of adolescent depression, an overview and cost-of-illness study. *Journal of Affective Disorders*, *241*, 256–262. <https://doi.org/10.1016/j.jad.2018.06.015>

Bolanis, D., Orri, M., Castellanos-Ryan, N., Renaud, J., Montreuil, T., Boivin, M., Vitaro, F., Tremblay, R. E., Turecki, G., Côté, S. M., Séguin, J. R., & Geoffroy, M.-C. (2020). Cannabis use, depression and suicidal ideation in adolescence: Direction of associations in a population based cohort. *Journal of Affective Disorders*, *274*, 1076–1083. <https://doi.org/10.1016/j.jad.2020.05.136>

Bonn-Miller, M. O., Boden, M. T., Bucossi, M. M., & Babson, K. A. (2014). Self-reported cannabis use characteristics, patterns and helpfulness among medical cannabis users. *The American Journal of Drug and Alcohol Abuse*, *40*(1), 23–30. <https://doi.org/10.3109/00952990.2013.821477>

Bottorff, J. L., Johnson, J. L., Moffat, B. M., & Mulvogue, T. (2009). Relief-oriented use of marijuana by teens. *Substance Abuse Treatment, Prevention, and Policy*, *4*. <https://doi.org/10.1186/1747-597X-4-7>

Bovasso, G. B. (2001). Cannabis abuse as a risk factor for depressive symptoms. *The American Journal of Psychiatry*, *158*(12), 2033–2037. <https://doi.org/10.1176/appi.ajp.158.12.2033>

Brière, F. N., Rohde, P., Seeley, J. R., Klein, D., & Lewinsohn, P. M. (2015). Adolescent suicide attempts and adult adjustment. *Depression and Anxiety*, *32*(4), 270–276. <https://doi.org/10.1002/da.22296>

Brook, D. W., Brook, J. S., Zhang, C., Cohen, P., & Whiteman, M. (2002). Drug use and the risk of major depressive disorder, alcohol dependence, and substance use disorders. *Archives of General Psychiatry*, *59*(11), 1039–1044. <https://doi.org/10.1001/archpsyc.59.11.1039>

Carvalho, A. F., Stubbs, B., Vancampfort, D., Kloiber, S., Maes, M., Firth, J., Kurdyak, P. A., Stein, D. J., Rehm, J., & Koyanagi, A. (2019). Cannabis use and suicide attempts among 86,254 adolescents aged 12-15 years from 21 low- and middle-income countries. *European Psychiatry: The Journal of the Association of European Psychiatrists*, *56*, 8–13. <https://doi.org/10.1016/j.eurpsy.2018.10.006>

Curtin, S. C. & Heron, M. (2019). Death Rates Due to Suicide and Homicide Among Persons Aged 10–24: United States, 2000–2017. *NCHS Data Brief* 2019; 352, 8. <https://www.cdc.gov/nchs/products/databriefs/db352.htm>.

Curtin S. C., Warner M., & Hedegaard H. (2016). Increase in Suicide in the United States, 1999—2014. *National Center for Health Statistics, ed. Vol NCHS data brief, no 241.* Hyattsville, MD; 2016.

<https://www.cdc.gov/nchs/products/databriefs/db241.htm>

Danielsson, A.-K., Lundin, A., Allebeck, P., & Agardh, E. (2016). Cannabis use and psychological distress: An 8-year prospective population-based study among Swedish men and women. *Addictive Behaviors*, *59*, 18–23. <https://doi.org/10.1016/j.addbeh.2016.03.005>

Degenhardt, L., Ferrari, A. J., Calabria, B., Hall, W. D., Norman, R. E., McGrath, J., Flaxman, A. D., Engell, R. E., Freedman, G. D., Whiteford, H. A., & Vos, T. (2013). The global epidemiology and contribution of cannabis use and dependence to the global burden of disease: Results from the GBD 2010 study. *PloS One*, *8*(10), e76635. <https://doi.org/10.1371/journal.pone.0076635>

Dillon FR, Turner CW, Robbins MS, Szapocznik J. Concordance among biological, interview, and self report measures of drug use among African American and Hispanic adolescents referred for drug abuse treatment. Psychology of addictive behaviors : journal of the Society of Psychologists in Addictive Behaviors. Dec 2005;19(4):404-13. doi:10.1037/0893-164x.19.4.404

Eckhoff, C., Sørvold, M. T., & Kvernmo, S. (2020). Adolescent self-harm and suicidal behavior and young adult outcomes in indigenous and non-indigenous people. *European Child & Adolescent Psychiatry*, *29*(7), 917–927. <https://doi.org/10.1007/s00787-019-01406-5>

Fergusson, D. M., Boden, J. M., & Horwood, L. J. (2007). Recurrence of major depression in adolescence and early adulthood, and later mental health, educational and economic outcomes. *The British Journal of Psychiatry: The Journal of Mental Science*, *191*, 335–342. <https://doi.org/10.1192/bjp.bp.107.036079>

Fergusson, D. M., Horwood, L. J., Ridder, E. M., & Beautrais, A. L. (2005). Suicidal behaviour in adolescence and subsequent mental health outcomes in young adulthood. *Psychological Medicine*, *35*(7), 983–993. <https://doi.org/10.1017/s0033291704004167>

Fergusson, D. M., Horwood, L. J., & Swain-Campbell, N. (2002). Cannabis use and psychosocial adjustment in adolescence and young adulthood. *Addiction (Abingdon, England)*, *97*(9), 1123–1135. <https://doi.org/10.1046/j.1360-0443.2002.00103.x>

Fergusson, D. M., & Woodward, L. J. (2002). Mental health, educational, and social role outcomes of adolescents with depression. *Archives of General Psychiatry*, *59*(3), 225–231. <https://doi.org/10.1001/archpsyc.59.3.225>

Fuhrmann, D., Knoll, L. J., & Blakemore, S.-J. (2015). Adolescence as a Sensitive  Period of Brain Development. *Trends in Cognitive Sciences*, *19*(10), 558–566. <https://doi.org/10.1016/j.tics.2015.07.008>

GBD 2017 Disease and Injury Incidence and Prevalence Collaborators. (2018). Global, regional, and national incidence, prevalence, and years lived with disability for 354 diseases and injuries for 195 countries and territories, 1990-2017: A systematic analysis for the Global Burden of Disease Study 2017. *Lancet (London, England)*, *392*(10159), 1789–1858. <https://doi.org/10.1016/S0140-6736(18)32279-7>

Giedd, J. N., Blumenthal, J., Jeffries, N. O., Castellanos, F. X., Liu, H., Zijdenbos, A., Paus, T., Evans, A. C., & Rapoport, J. L. (1999). Brain development during childhood and adolescence: A longitudinal MRI study. *Nature Neuroscience*, *2*(10), 861–863. <https://doi.org/10.1038/13158>

Glied, S., & Pine, D. S. (2002). Consequences and Correlates of Adolescent Depression. *Archives of Pediatrics & Adolescent Medicine*, *156*(10), 1009. <https://doi.org/10.1001/archpedi.156.10.1009>

Gobbi, G., Atkin, T., Zytynski, T., Wang, S., Askari, S., Boruff, J., Ware, M., Marmorstein, N., Cipriani, A., Dendukuri, N., & Mayo, N. (2019). Association of Cannabis Use in Adolescence and Risk of Depression, Anxiety, and Suicidality in Young Adulthood. *JAMA Psychiatry*, *76*(4), 426–434. <https://doi.org/10.1001/jamapsychiatry.2018.4500>

Gorzalka, B. B., & Hill, M. N. (2011). Putative role of endocannabinoid signaling in the etiology of depression and actions of antidepressants. *Progress in Neuro-Psychopharmacology & Biological Psychiatry*, *35*(7), 1575–1585. <https://doi.org/10.1016/j.pnpbp.2010.11.021>

Gryczynski, J., Mitchell, S. G., Schwartz, R. P., Kelly, S. M., Dušek, K., Monico, L., O’Grady, K. E., Brown, B. S., Oros, M., & Hosler, C. (2019). Disclosure of Adolescent Substance Use in Primary Care: Comparison of Routine Clinical Screening and Anonymous Research Interviews. *The Journal of Adolescent Health: Official Publication of the Society for Adolescent Medicine*, *64*(4), 541–543. <https://doi.org/10.1016/j.jadohealth.2018.10.009>

Hamilton, C. M., Strader, L. C., Pratt, J. G., Maiese, D., Hendershot, T., Kwok, R. K., Hammond, J. A., Huggins, W., Jackman, D., Pan, H., Nettles, D. S., Beaty, T. H., Farrer, L. A., Kraft, P., Marazita, M. L., Ordovas, J. M., Pato, C. N., Spitz, M. R., Wagener, D., … Haines, J. (2011). The PhenX Toolkit: Get the most from your measures. *American Journal of Epidemiology*, *174*(3), 253–260. <https://doi.org/10.1093/aje/kwr193>

Hengartner, M. P., Angst, J., Ajdacic-Gross, V., & Rössler, W. (2020). Cannabis use during adolescence and the occurrence of depression, suicidality and anxiety disorder across adulthood: Findings from a longitudinal cohort study over 30 years. *Journal of Affective Disorders*, *272*, 98–103. <https://doi.org/10.1016/j.jad.2020.03.126>

Heron, M. (2019). Deaths: Leading Causes for 2017. *National Vital Statistics Reports: From the Centers for Disease Control and Prevention, National Center for Health Statistics, National Vital Statistics System*, *68*(6), 1–77.

Hill, M. N., & Gorzalka, B. B. (2009). Impairments in endocannabinoid signaling and depressive illness. *JAMA*, *301*(11), 1165–1166. <https://doi.org/10.1001/jama.2009.369>

Hirvonen, J., Goodwin, R. S., Li, C.-T., Terry, G. E., Zoghbi, S. S., Morse, C., Pike, V. W., Volkow, N. D., Huestis, M. A., & Innis, R. B. (2012). Reversible and regionally selective downregulation of brain cannabinoid CB1 receptors in chronic daily cannabis smokers. *Molecular Psychiatry*, *17*(6), 642–649. <https://doi.org/10.1038/mp.2011.82>

Huas, C., Hassler, C., & Choquet, M. (2008). Has occasional cannabis use among adolescents also to be considered as a risk marker? *European Journal of Public Health*, *18*(6), 626–629. <https://doi.org/10.1093/eurpub/ckn065>

Kamenetz, A. (2020, September 10). *The Pandemic Has Researchers Worried About Teen Suicide*. <https://www.wbur.org/npr/911117577/the-pandemic-has-researchers-worried-about-teen-suicide>

Kandel, D. B., Raveis, V. H., & Davies, M. (1991). Suicidal ideation in adolescence: Depression, substance use, and other risk factors. *Journal of Youth and Adolescence*, *20*(2), 289–309. <https://doi.org/10.1007/BF01537613>

Kann, L. (2018). Youth Risk Behavior Surveillance—United States, 2017. *MMWR. Surveillance Summaries*, *67*. <https://doi.org/10.15585/mmwr.ss6708a1>

Kessler, R. C., Berglund, P., Demler, O., Jin, R., Merikangas, K. R., & Walters, E. E. (2005). Lifetime prevalence and age-of-onset distributions of DSM-IV disorders in the National Comorbidity Survey Replication. *Archives of General Psychiatry*, *62*(6), 593–602. <https://doi.org/10.1001/archpsyc.62.6.593>

Lim, K. X., Rijsdijk, F., Hagenaars, S. P., Socrates, A., Choi, S. W., Coleman, J. R. I., Glanville, K. P., Lewis, C. M., & Pingault, J.-B. (2020). Studying individual risk factors for self-harm in the UK Biobank: A polygenic scoring and Mendelian randomisation study. *PLoS Medicine*, *17*(6), e1003137. <https://doi.org/10.1371/journal.pmed.1003137>

Lynskey, M. T., Glowinski, A. L., Todorov, A. A., Bucholz, K. K., Madden, P. A. F., Nelson, E. C., Statham, D. J., Martin, N. G., & Heath, A. C. (2004). Major depressive disorder, suicidal ideation, and suicide attempt in twins discordant for cannabis dependence and early-onset cannabis use. *Archives of General Psychiatry*, *61*(10), 1026–1032. <https://doi.org/10.1001/archpsyc.61.10.1026>

Manrique-Garcia, E., Zammit, S., Dalman, C., Hemmingsson, T., & Allebeck, P. (2012). Cannabis use and depression: A longitudinal study of a national cohort of Swedish conscripts. *BMC Psychiatry*, *12*, 112. <https://doi.org/10.1186/1471-244X-12-112>

Mars, B., Heron, J., Klonsky, E. D., Moran, P., O’Connor, R. C., Tilling, K., Wilkinson, P., & Gunnell, D. (2019). Predictors of future suicide attempt among adolescents with suicidal thoughts or non-suicidal self-harm: A population-based birth cohort study. *The Lancet Psychiatry*, *6*(4), 327–337. <https://doi.org/10.1016/S2215-0366(19)30030-6>

Miech RA, Schulenberg JE, Johnston LD, Bachman JG, O'Malley PM, Patrick ME. (2019). National Adolescent Drug Trends in 2019: Findings Released. http://www.monitoringthefuture.org. Accessed 08/21/2020.

Miranda, R., Ortin, A., Scott, M., & Shaffer, D. (2014). Characteristics of suicidal ideation that predict the transition to future suicide attempts in adolescents. *Journal of Child Psychology and Psychiatry, and Allied Disciplines*, *55*(11), 1288–1296. <https://doi.org/10.1111/jcpp.12245>

Moitra, E., Christopher, P. P., Anderson, B. J., & Stein, M. D. (2015). Coping-motivated marijuana use correlates with DSM-5 cannabis use disorder and psychological distress among emerging adults. *Psychology of Addictive Behaviors: Journal of the Society of Psychologists in Addictive Behaviors*, *29*(3), 627–632. <https://doi.org/10.1037/adb0000083>

The National Institute of Mental Health. (2020). U.S. Leading Categories of Diseases/Disorders. <https://www.nimh.nih.gov/health/statistics/disability/us-leading-categories-of-diseases-disorders.shtml>

Nock, M. K., Borges, G., Bromet, E. J., Cha, C. B., Kessler, R. C., & Lee, S. (2008). Suicide and suicidal behavior. *Epidemiologic Reviews*, *30*, 133–154. <https://doi.org/10.1093/epirev/mxn002>

Nock, M. K., Holmberg, E. B., Photos, V. I., & Michel, B. D. (2007). Self-Injurious Thoughts and Behaviors Interview: Development, reliability, and validity in an adolescent sample. *Psychological Assessment*, *19*(3), 309–317. <https://doi.org/10.1037/1040-3590.19.3.309>

Nock, M. K., Kleiman, E. M., Abraham, M., Bentley, K. H., Brent, D. A., Buonopane, R. J., Castro‐Ramirez, F., Cha, C. B., Dempsey, W., Draper, J., Glenn, C. R., Harkavy‐Friedman, J., Hollander, M. R., Huffman, J. C., Lee, H. I. S., Millner, A. J., Mou, D., Onnela, J., Picard, R. W., … Pearson, J. L. (2020). Consensus Statement on Ethical & Safety Practices for Conducting Digital Monitoring Studies with People at Risk of Suicide and Related Behaviors. *Psychiatric Research and Clinical Practice*, *3*(2), 57–66. <https://doi.org/10.1176/appi.prcp.20200029>

Orri, M., Séguin, J. R., Castellanos-Ryan, N., Tremblay, R. E., Côté, S. M., Turecki, G., & Geoffroy, M.-C. (2020). A genetically informed study on the association of cannabis, alcohol, and tobacco smoking with suicide attempt. *Molecular Psychiatry*. <https://doi.org/10.1038/s41380-020-0785-6>

Patton, G. C., Coffey, C., Carlin, J. B., Degenhardt, L., Lynskey, M., & Hall, W. (2002). Cannabis use and mental health in young people: Cohort study. *BMJ : British Medical Journal*, *325*(7374), 1195–1198.

Pistis, M., Perra, S., Pillolla, G., Melis, M., Muntoni, A. L., & Gessa, G. L. (2004). Adolescent exposure to cannabinoids induces long-lasting changes in the response to drugs of abuse of rat midbrain dopamine neurons. *Biological Psychiatry*, *56*(2), 86–94. <https://doi.org/10.1016/j.biopsych.2004.05.006>

Posner, K., Brown, G. K., Stanley, B., Brent, D. A., Yershova, K. V., Oquendo, M. A., Currier, G. W., Melvin, G. A., Greenhill, L., Shen, S., & Mann, J. J. (2011). The Columbia-Suicide Severity Rating Scale: Initial validity and internal consistency findings from three multisite studies with adolescents and adults. *The American Journal of Psychiatry*, *168*(12), 1266–1277. <https://doi.org/10.1176/appi.ajp.2011.10111704>

Poulin, C., Hand, D., Boudreau, B., & Santor, D. (2005). Gender differences in the association between substance use and elevated depressive symptoms in a general adolescent population. *Addiction (Abingdon, England)*, *100*(4), 525–535. <https://doi.org/10.1111/j.1360-0443.2005.01033.x>

Rasic, D., Weerasinghe, S., Asbridge, M., & Langille, D. B. (2013). Longitudinal associations of cannabis and illicit drug use with depression, suicidal ideation and suicidal attempts among Nova Scotia high school students. *Drug and Alcohol Dependence*, *129*(1–2), 49–53. <https://doi.org/10.1016/j.drugalcdep.2012.09.009>

Reddy, M. S. (2010). Depression: The Disorder and the Burden. *Indian Journal of Psychological Medicine*, *32*(1), 1–2. <https://doi.org/10.4103/0253-7176.70510>

Rey, J. M., Sawyer, M. G., Raphael, B., Patton, G. C., & Lynskey, M. (2002). Mental health of teenagers who use cannabis. Results of an Australian survey. *The British Journal of Psychiatry: The Journal of Mental Science*, *180*, 216–221. <https://doi.org/10.1192/bjp.180.3.216>

Reynolds, S. K., Lindenboim, N., Comtois, K. A., Murray, A., & Linehan, M. M. (2006). Risky assessments: Participant suicidality and distress associated with research assessments in a treatment study of suicidal behavior. *Suicide & Life-Threatening Behavior*, *36*(1), 19–34. <https://doi.org/10.1521/suli.2006.36.1.19>

Roditis, M. L., & Halpern-Felsher, B. (2015). Adolescents’ Perceptions of Risks and Benefits of Conventional Cigarettes, E-Cigarettes, and Marijuana: A Qualitative Analysis. *The Journal of Adolescent Health : Official Publication of the Society for Adolescent Medicine*, *57*(2), 179–185. <https://doi.org/10.1016/j.jadohealth.2015.04.002>

*Roberts DM, Schade MM, Mathew GM, Gartenberg D, Buxton OM. Detecting sleep using heart rate*

*and motion data from multisensor consumer-grade wearables, relative to wrist actigraphy and*

*polysomnography. Sleep. Jul 13 2020;43(7)doi:10.1093/sleep/zsaa045*

Rubino, T., & Parolaro, D. (2016). The Impact of Exposure to Cannabinoids in Adolescence: Insights From Animal Models. *Biological Psychiatry*, *79*(7), 578–585. <https://doi.org/10.1016/j.biopsych.2015.07.024>

Schafer, J. L., & Graham, J. W. (2002). Missing data: Our view of the state of the art. *Psychological Methods*, *7*(2), 147–177. <https://doi.org/10.1037/1082-989X.7.2.147>

Sellers, R., Warne, N., Pickles, A., Maughan, B., Thapar, A., & Collishaw, S. (2019). Cross‐cohort change in adolescent outcomes for children with mental health problems. *Journal of Child Psychology and Psychiatry, and Allied Disciplines*, *60*(7), 813–821. <https://doi.org/10.1111/jcpp.13029>

Sheehan, D. V., Sheehan, K. H., Shytle, R. D., Janavs, J., Bannon, Y., Rogers, J. E., Milo, K. M., Stock, S. L., & Wilkinson, B. (2010). Reliability and validity of the Mini International Neuropsychiatric Interview for Children and Adolescents (MINI-KID). The Journal of clinical psychiatry, 71(3), 313–326. <https://doi.org/10.4088/JCP.09m05305whi>

Shiffman, S., Scholl, S. M., Mao, J., Ferguson, S. G., Hedeker, D., & Tindle, H. A. (2020). Ecological momentary assessment of temptations and lapses in non-daily smokers. *Psychopharmacology*, *237*(8), 2353–2365. <https://doi.org/10.1007/s00213-020-05539-3>

Silins, E., Horwood, L. J., Patton, G. C., Fergusson, D. M., Olsson, C. A., Hutchinson, D. M., Spry, E., Toumbourou, J. W., Degenhardt, L., Swift, W., Coffey, C., Tait, R. J., Letcher, P., Copeland, J., Mattick, R. P., & Cannabis Cohorts Research Consortium. (2014). Young adult sequelae of adolescent cannabis use: An integrative analysis. *The Lancet. Psychiatry*, *1*(4), 286–293. <https://doi.org/10.1016/S2215-0366(14)70307-4>

Slap, G. B., Vorters, D. F., Chaudhuri, S., & Centor, R. M. (1989). Risk factors for attempted suicide during adolescence. *Pediatrics*, *84*(5), 762–772.

Sobell, M. B., Sobell, L. C., Klajner, F., Pavan, D., & Basian, E. (1986). The reliability of a timeline method for assessing normal drinker college students’ recent drinking history: Utility for alcohol research. *Addictive Behaviors*, *11*(2), 149–161. <https://doi.org/10.1016/0306-4603(86)90040-7>

Spirito, A., Brown, L., Overholser, J., & Fritz, G. (1989). Attempted suicide in adolescence: A review and critique of the literature. *Clinical Psychology Review*, *9*(3), 335–363. <https://doi.org/10.1016/0272-7358(89)90061-5>

Swahn, M. H., Bossarte, R. M., Choquet, M., Hassler, C., Falissard, B., & Chau, N. (2012). Early substance use initiation and suicide ideation and attempts among students in France and the United States. *International Journal of Public Health*, *57*(1), 95–105. <https://doi.org/10.1007/s00038-011-0255-7>

Tiet, Q. Q., Bird, H. R., Davies, M., Hoven, C., Cohen, P., Jensen, P. S., & Goodman, S. (1998). Adverse life events and resilience. *Journal of the American Academy of Child and Adolescent Psychiatry*, *37*(11), 1191–1200. <https://doi.org/10.1097/00004583-199811000-00020>

Volkow, N. D., Baler, R. D., Compton, W. M., & Weiss, S. R. B. (2014). Adverse Health Effects of Marijuana Use. *The New England Journal of Medicine*, *370*(23), 2219–2227. <https://doi.org/10.1056/NEJMra1402309>

Wenzel, A., Berchick, E. R., Tenhave, T., Halberstadt, S., Brown, G. K., & Beck, A. T. (2011). Predictors of suicide relative to other deaths in patients with suicide attempts and suicide ideation: A 30-year prospective study. *Journal of Affective Disorders*, *132*(3), 375–382. <https://doi.org/10.1016/j.jad.2011.03.006>

Werner, K. B., McCutcheon, V. V., Agrawal, A., Sartor, C. E., Nelson, E. C., Heath, A. C., & Bucholz, K. K. (2016). The association of specific traumatic experiences with cannabis initiation and transition to problem use: Differences between African-American and European-American women. *Drug and Alcohol Dependence*, *162*, 162–169. <https://doi.org/10.1016/j.drugalcdep.2016.03.003>

Worden, B. L., Bowe, W. M., & Tolin, D. F. (2017). An open trial of cognitive behavioral therapy with contingency management for hoarding disorder. *Journal of Obsessive-Compulsive and Related Disorders*, *12*, 78–86. <https://doi.org/10.1016/j.jocrd.2016.12.005>

World Health Organization. Suicide. (2019). <https://www.who.int/news-room/fact-sheets/detail/suicide>.
